# Transcriptomics analysis of hepatotoxicity induced by the pesticides imazalil, thiacloprid and clothianidin alone or in binary mixtures in a 28-day study in female Wistar rats

**DOI:** 10.1007/s00204-020-02969-y

**Published:** 2021-01-11

**Authors:** Jimmy Alarcan, Heike Sprenger, Julia Waizenegger, Dajana Lichtenstein, Claudia Luckert, Philip Marx-Stoelting, Alfonso Lampen, Albert Braeuning

**Affiliations:** 1 Department of Food Safety, German Federal Institute for Risk Assessment, Max-Dohrn-Straße 8-10, 10589 Berlin, Germany; 2 Department of Pesticides Safety, German Federal Institute for Risk Assessment, Max-Dohrn-Straße 8-10, 10589 Berlin, Germany

**Keywords:** Pesticides, Mixture effects, Transcriptomics, EuroMix

## Abstract

Co-occurrence of pesticide residues in food commodities raises a potential safety issue as their mixture effects on human health are largely unknown. In a previous study, we reported the toxicological effects (pathology and histopathology) of imazalil (IMZ), thiacloprid (THI), and clothianidin (CTD) alone and in binary mixtures in a 28-day oral gavage study in female Wistar rats. Five dose levels (up to 350 mg/kg body weight/day) ranging from a typical toxicological reference value to a clear effect dose were applied. In the present study, we undertook a transcriptomics analysis of rat livers by means of total RNA sequencing (RNA-Seq). Bioinformatic data analysis involving Ingenuity Pathway Analysis (IPA) was used to gain mechanistic information on hepatotoxicity-related pathways affected after treatment with the pesticides, alone and in mixtures. Our data show that 2986 genes were differentially regulated by CTD while IMZ and THI had effects on 194 and 225 genes, respectively. All three individual compounds shared a common subset of genes whose network is associated with xenobiotic metabolism and nuclear receptor activation. Similar networks were retrieved for the mixtures. Alterations in the expression of individual genes were in line with the assumption of dose addition. Our results bring new insight into the hepatotoxicity mechanisms of IMZ, THI, and CTD and their mixtures.

**Supplementary Information:**

The online version contains supplementary material available at 10.1007/s00204-020-02969-y.

## Introduction

Humans are exposed to multiple pesticide residues through the environment or their daily diet (Cataudella et al. 2012; Cedergreen 2014; Kortenkamp 2014). Facing the need to integrate this stake, strategies have been proposed and implemented to help with the risk assessment of chemical mixtures (EFSA 2013; OECD 2018; Rotter et al. 2018). One critical aspect in the study of chemical mixtures is the identification of modes of action (MoA) underlying the toxicological outcomes. Indeed, such identification not only helps to classify substances into groups [i.e., cumulative assessment groups (CAG)], but is also important when using most mathematical models for modeling of mixture effects (Cedergreen 2014; EFSA 2013; Kortenkamp et al. 2009).

Furthermore, the integration of knowledge about the toxicological MoA in the risk assessment of substances has gained increasing attention as the development of tools such as the concept of adverse outcome pathways (AOP) emerged (EFSA 2014; Martens et al. 2018; Perkins et al. 2019). However, establishing biological pathways linking changes at the molecular level to the observed outcome at the physio-pathological level represents a scientific challenge that single endpoint investigation cannot face efficiently. In the last decade, the rapid development and implementation of omics technologies has enabled the possibility of characterization and quantification of large sets of biomolecules in a single run. Such approaches allow for better understanding and deciphering of the MoA of a given substance and in the context of chemical mixtures could help to identify potential biomarkers of mixture toxicity (Altenburger et al. 2012; Martins et al. 2019; Marx-Stoelting et al. 2015; Seeger et al. 2019).

We have recently published the outcome of a 28-day oral study in female Wistar rats with the three pesticidal active compounds imazalil (IMZ), thiacloprid (THI) and clothianidin (CTD) (Alarcan et al. 2020). Those substances have been assigned to the CAG liver toxicity and recent exposure studies revealed a predominant occurrence in food or even in human urine samples, thus raising a concern about safety (Craddock et al. 2019; Crepet et al. 2019; Nielsen et al. 2012). The pesticides were either being administrated alone or in binary combinations, using an equipotency-based approach. Reported effects included an increase in liver weight, hepatocellular hypertrophy and cytoplasmic changes of the hepatocytes. Additionally, liver and kidney residue analysis showed alterations in pesticide residues when applied in mixtures as compared to the levels of pesticide residues for the single compound treatment (Alarcan et al. 2020).

Even after having documented the toxicological effects at the histopathological level, the mechanisms by which IMZ, THI and CTD induce adverse effects at the molecular level are still to be characterized. Using transfected human HepG2 liver cells, we previously showed the activation of human pregnane X receptor (PXR) and constitutive androstane receptor (CAR) by IMZ in a dual luciferase-based transactivation assay (Lichtenstein et al. 2020). Furthermore, in this study, THI was found to activate PXR and peroxisome proliferator-activated receptor γ (PPARγ) while CTD inhibited PPARα. These findings on PXR and CAR activation were complemented by the observation of up-regulation in the expression of associated target genes (e.g., CYP genes) in human HepaRG hepatocarcinoma cells (Lichtenstein et al. 2020). Of note, an increase in CYP content in rats following 30-day treatment with THI was also reported by others (Hendawi et al. 2016).

The present study constitutes a follow-up to the previous publication of pesticide hepatotoxicity in vivo (Alarcan et al. 2020) and aims to investigate the molecular effects of pesticides in rat liver, both individually and in combination. To this end, we undertook a transcriptomics analysis using total RNA sequencing (RNA-Seq). Furthermore, gene ontology (GO) term enrichment and ingenuity pathway analysis (IPA) were performed to get insight into the molecular MoA of a compound by comparing and retrieving datasets of genes that are associated with a specific biological function or pathway (Krämer et al. 2014). The present study is part of the EuroMix project (European Test and Risk Assessment Strategies for Mixtures—information available on www.euromixproject.eu).

## Materials and methods

### Test substances

Clothianidin (CAS no. 210880-92-5, Batch no. EDFL036131) and thiacloprid (CAS no. 111988-49-9, Batch no. EDTE013890) were supplied by Bayer AG (Leverkusen, Germany). Imazalil (CAS no. 35554-44-0, Batch no. 1-TAH-30-1) was obtained from Sigma-Aldrich (Taufkirchen, Germany).

### Animals and treatment

Detailed description of study design can be found in Alarcan et al. (2020). Briefly, young adult female Wistar rats (Crl:WI) were acclimatized to the laboratory conditions for at least 1 week and were weighed and monitored for their health status. Female rats were chosen considering a previous 28-day study in which only female rats showed increase in liver weight following IMZ administration [study from “Draft Assessment Report on the Active Substance Imazalil” (DAR)]. Rats were allocated in a randomized manner to the different treatment groups (see Table 1) and were maintained under conventional laboratory conditions: constant 12/12-h light/dark cycle with controlled temperature at 22 °C ± 2 °C and humidity at 55% ± 15%. The specified doses of the test compounds were daily administered to the rats by oral gavage for 28 days. Test compounds were suspended in 0.5% aqueous carboxymethyl-cellulose with a dosing volume of 7.5 ml/kg bw. Rats of the control group were treated with the vehicle only (0.5% aqueous carboxymethyl-cellulose in deionized water). Dose selection was based on studies from DARs of the individual compounds. Equipotent mixtures were established following the relative potency factors (RPF) approach. The determined maximal doses of the single compounds were used to calculate provisional RPF: RPF _CTD/THI_ = 2.50, RPF _CTD/IMZ_ = 2.92, and RPF _THI/IMZ_ = 1.17.

**Table 1:** Study design and dosing scheme of the 28-day oral toxicity study in female Wistar rats

Group	Treatment	Dose mg/kg bw/day		Number of rats
1	Control (vehicle)	–	–	8
2	Thiacloprid	Very low	10	4
3	Thiacloprid	Low	43	4
4	Thiacloprid	Mid	75	4
5	Thiacloprid	High	108	4
6	Thiacloprid	Very high	140	3*
7	Imazalil	Very low	10	4
8	Imazalil	Low	38	4
9	Imazalil	Mid	65	4
10	Imazalil	High	93	4
11	Imazalil	Very high	120	3*
12	Clothianidin	Very low	100	4
13	Clothianidin	Low	163	4
14	Clothianidin	Mid	225	4
15	Clothianidin	High	288	3*
16	Clothianidin	Very high	350	2*
17	Thiacloprid + Imazalil	Very low	5/4	4
18	Thiacloprid + Imazalil	Low	21/18	4
19	Thiacloprid + Imazalil	Mid	38/32	4
20	Thiacloprid + Imazalil	High	54/46	4
21	Thiacloprid + Imazalil	Very high	70/60	4
22	Thiacloprid + Clothianidin	Very low	5/13	4
23	Thiacloprid + Clothianidin	Low	21/53	4
24	Thiacloprid + Clothianidin	Mid	38/94	4
25	Thiacloprid + Clothianidin	High	54/134	4
26	Thiacloprid + Clothianidin	Very high	70/175	3*
27	Imazalil + Clothianidin	Very low	5/15	4
28	Imazalil + Clothianidin	Low	19/55	4
29	Imazalil + Clothianidin	Mid	33/95	4
30	Imazalil + Clothianidin	High	46/135	4
31	Imazalil + Clothianidin	Very high	60/175	4

The asterisk indicates that one or more rats were killed in moribund condition (for IMZ, the rat was found dead)

After sacrifice, the liver was isolated and prepared as follows: the liver hilus as well as the adjacent connective tissue were prepared off and discarded, and the liver was trimmed into the individual liver lobes. Lobes were cut into small cubes (approx. 5 × 5 mm), cleansed on filter paper, immediately shock frozen in liquid nitrogen, and stored at − 80 °C until further analysis.

### RNA preparation

Total RNA was isolated from approximately 20–40 mg frozen rat tissue sample using the RNeasy Mini Kit (Qiagen, Hilden, Germany) following the manufacturer’s protocol. The quality and concentration of RNA was determined using NanoQuant plate with Infinite M200 Pro plate reader (Tecan group, Männedorf, Switzerland) following the instruction manual and the integrity of RNA was evaluated using the Agilent RNA 6000 Nano LabChip kit together with the Agilent 2100 Bioanalyzer (Agilent, Santa Clara, USA) according to the manufacturer’s protocol. All RNA samples had RNA integrity numbers (RINs) greater than 9.3. For each sample, one half was used for total RNA sequencing while the other half was used for real-time quantitative PCR analysis. Samples obtained from the animals of each treatment group represented independent biological replicates.

### Total RNA sequencing

RNA-Seq was done only for the rats of the high dose groups. For sequencing analysis, the samples were shipped to CeGaT GmbH (Tübingen, Germany) for library preparation using TruSeq Stranded Total RNA Sample Preparation-Kit (Illumina) from 100 ng RNA per sample. Sequencing was performed using the Illumina NovaSeq6000 platform. Depths of ~ 50–160 million paired-end 100 bp reads were generated for each sample (see Supplementary Table S1 for details). The raw RNA sequencing data are available from GEO under accession number GSE153986. Adapters were trimmed by Skewer version 0.2.2 (Jiang et al. 2014) and data quality was assessed by FastQC version 0.11.5 (Andrews 2010). Reads were mapped to the rat reference genome Rnor_5.0 and counted per gene ID using STAR version 2.5.2b (Dobin et al. 2013).

### Statistical analysis

After removing genes with low expression (sum of reads across all samples below two), retained genes were analyzed by the R-package (R Core Team 2013) DESeq2 version 1.15.1 (Love et al. 2014) using default settings estimation of size factors and dispersion. Negative Binomial GLM fitting and Wald statistics were applied to test for differential gene expression between each treatment and control conditions, respectively. False discovery rate (FDR) was used to control for multiple testing (Benjamini and Hochberg 1995). Only genes with a *q*-value < 0.05 were included in the further analyses. Variance stabilizing transformation was applied prior to probabilistic PCA (ppca) on pareto scaled and centered data by the R-package pcaMethods (Stacklies et al. 2007). K-means clustering of transformed and scaled gene expression data with 12 centers was performed by the R-package stats. Theoretical log_2_-fold change values (LFC) for mixtures were computed as a combination of single compounds adjusting for the dose: ${LFC}_{IMZ+CTD}={0.5\times LFC}_{IMZ}+{0.47\times LFC}_{CTD}$ (the factor for CTD results from the division of 135 mg/kg bw/day in mixture by 288 mg/kg bw/day in single compound treatment).

### Verification of target genes by real-time quantitative PCR analysis

A quantity of 1000 ng RNA were reverse transcribed into cDNA using High Capacity cDNA Reverse Transcription Kit (Applied Biosystems, Darmstadt, Germany) and thermal cycling conditions were chosen according to the manufacturer’s protocol. The primers were designed with the free webtool Primer3. All primers (Supplementary Table S2) were purchased from Eurofins (Luxemburg). Real-time quantitative PCR (qRT-PCR) was performed on ABI 7900 HT Fast Real-Time PCR system instrument (Applied Biosystems, Darmstadt, Germany) using Maxima SYBR Green/Rox qPCR Mastermix (Life Technologies, Carlsbad, USA) with 5 μM primers and 1 µl cDNA in a total volume of 10 µl. Thermal cycling conditions were as follows: initial denaturation at 95 °C for 10 min, 40 cycles of denaturation at 95 °C for 15 s and combined annealing and elongation at 60 °C for 1 min, 15 s at 95 °C and final elongation at 60 °C for 15 min. At the end of the run, a dissociation curve analysis was performed. For the relative quantification of mRNA content according to the 2^−∆∆Ct^ method (Livak and Schmittgen 2001), C_t_ values were normalized to *Actb* (encoding β-actin) and referred to vehicle-treated rats. Differences between means were determined by the nonparametric Kruskal–Wallis test followed by Dunn’s test (*p < 0.05; **p < 0.01; ***p < 0.001).

### Mixture analysis

The software PROAST (www.proast.nl) was used to investigate, whether the observed mixture effects comply with the assumption of dose addition. A detailed explanation on this statistical dose–response modeling can be found in previous studies (EPA 2012; Kienhuis et al. 2015). Briefly, the dose response curves obtained from the single compounds and from the compounds applied in mixture were plotted and their curve fit was estimated. In case the mixture deviates from the dose addition assumption, the plot of the mixture will shift to the left (synergism) or to the right (antagonism) in contrast to the plot of the single compounds.

### Ingenuity pathway analysis

Data were analyzed through the use of QIAGEN’s Ingenuity Pathway Analysis (IPA, QIAGEN Redwood City, www.qiagen.com/ingenuity; release 2014-06-24). We performed an upstream regulator analysis to determine transcription regulators potentially activated or inhibited. The right-tailed Fisher’s exact test was used to estimate the probability of association of a set of genes in the dataset with a transcription regulator by random chance alone. A *p* value < 0.05 was chosen as significance level. Results were filtered to include only ligand-dependent nuclear receptors and transcription regulators. The *p* value was used to evaluate the statistical significance of the overlap between the dataset genes and the genes which are regulated by the transcription regulator. The IPA *z*-score algorithm was used to identify transcription regulators that are expected to be activated or inhibited. A *z*-score ≥ 2 or ≤ − 2 predicts a significantly activated or inhibited transcription regulator state, respectively. Heatmap presentations were generated using matrix2png (Pavlidis and Noble 2003).

### GO term enrichment analysis

Gene ontology (GO) Biological Process term enrichment analysis was performed using a hypergeometric test by the R-package clusterProfiler version 3.14.3 (Yu et al. 2012). False discovery rate (FDR) was applied to control for multiple testing (Benjamini and Hochberg 1995) and an adjusted *p* value < 0.05 was considered as significant. Resulting GO terms were clustered by semantic similarity using Wang method and the R-package GOSemSim version 2.14.0 (Yu et al. 2010) and –log_10_(*p* value) were visualized in heatmaps using the R-package pheatmap version 1.0.12. The GO terms shown in heatmaps were filtered by removing redundant entries measured by semantic similarity (the complete results are shown in Supplementary Table S4–S6).

## Results

### Total RNA sequencing

To obtain in-depth information on the hepatic molecular MoA of pesticides alone and in mixtures, RNA-Seq was performed on the liver samples from the high dose group treatment. On average, 85% of the reads were mapped to the reference genome Rnor_5.0 (Supplementary Table S1). After filtering for genes with low expression, 19,904 genes were retained representing 75% of all annotated genes.

**Fig. 1:**
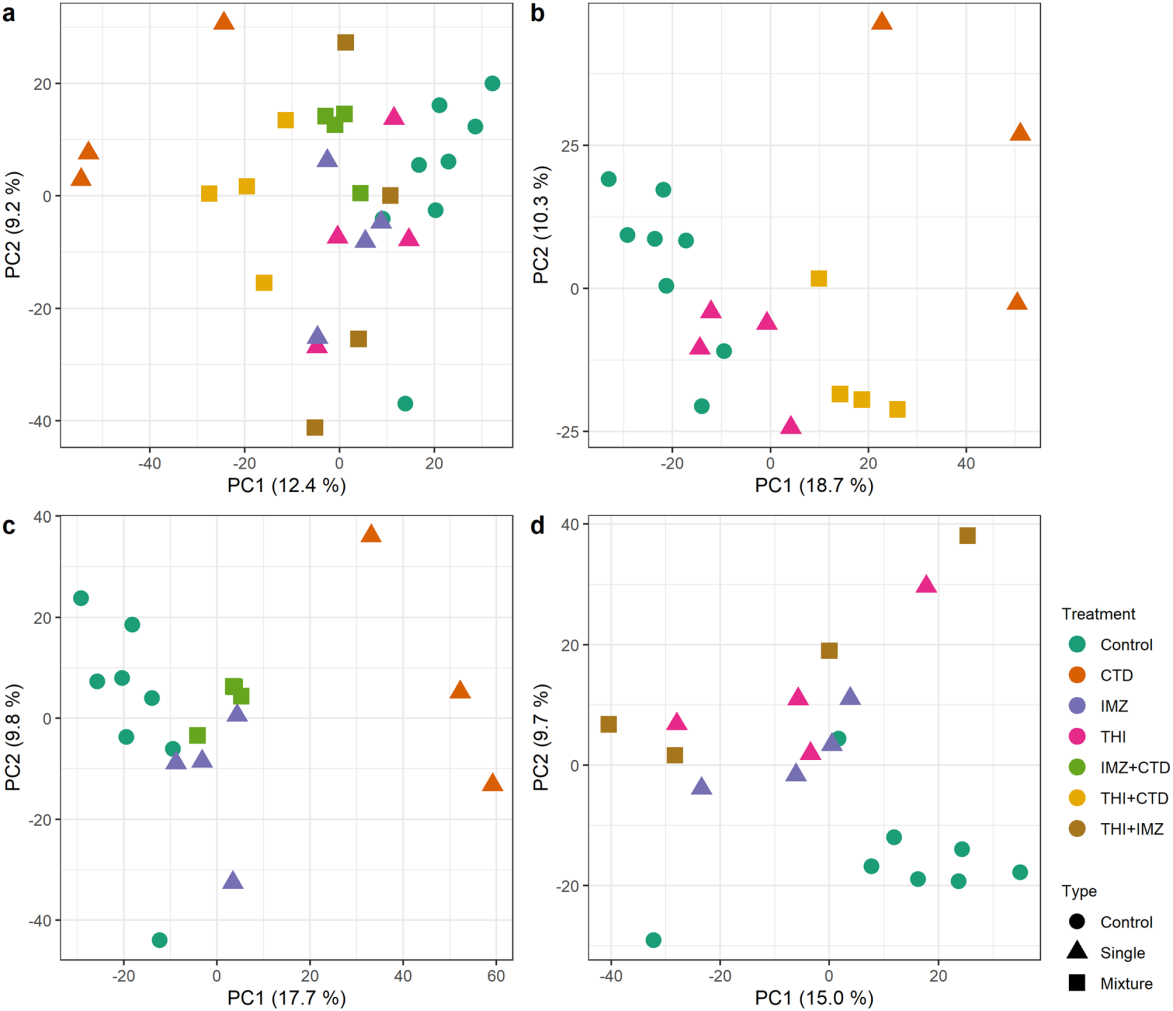
Principal component analysis (PCA) of transcriptome profiles of rat livers treated with different pesticidal active compounds alone or in mixtures was performed on normalized variance stabilized read counts. **a** Scores plot of samples for all conditions. PCA scores plots for data subsets of each mixture and the respective single compound for THI-CTD (**b**), IMZ + CTD (**c**) and THI + IMZ (**d**). Each treatment is indicated by a different color and the type of treatment by symbols

Principal component analysis (PCA) was employed to get an overall picture of the transcriptome profile upon pesticide treatment. As shown in Fig. 1a, the treatment effect contributed mainly to the transcriptome variation as PC1 explains 12.4% of the variation. PCA of subsets indicate a clear separation of control samples from the treated samples, especially for CTD (Fig. 1b–d). However, the effect of single treatment with THI (Fig. 1b) and IMZ (Fig. 1c) appeared to be less pronounced since these samples cluster together with control samples. Interestingly, the treatment with THI + CTD resulted in a gene expression pattern between the respective single compounds THI (Fig. 1b), while IMZ + CTD samples resemble rather the samples treated with IMZ alone (Fig. 1c). The samples of the mixture THI + IMZ clustered together with both single compounds indicating a comparable treatment effect on the transcriptome (Fig. 1d).

**Fig. 2:**
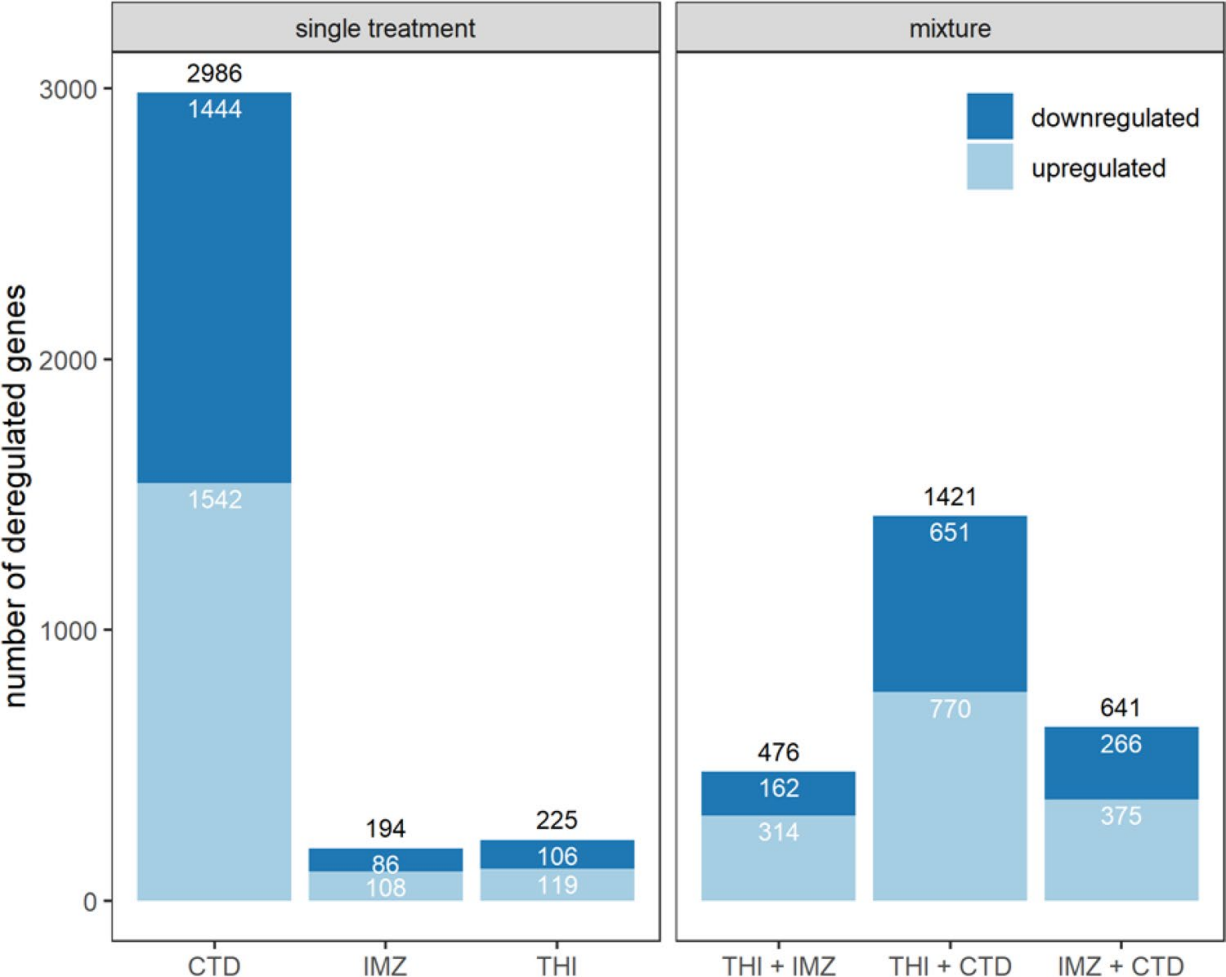
Bar plot of the number of DEG per single compound and mixture treatment in comparison to control conditions. Genes were filtered based on a *q*-value < 0.05

Analysis of differentially expressed genes (DEGs) was performed to assess the treatment effect of single compounds and mixtures in comparison to control conditions. In single treatment, CTD modulated the expression of 2986 genes while IMZ and THI differentially regulated 194 and 225 genes, respectively (Fig. 2). For all three compounds, repartition between up-regulated and down-regulated genes was well balanced. In mixture, THI + CTD modulated 1421 genes while IMZ + CTD and THI + IMZ modified the expression of 641 and 476 genes, respectively. Overall, the DEG numbers reflected the clustering of samples in the PCA plots (Fig. 1) where CTD treatment caused the strongest separation from control conditions.

**Fig. 3:**
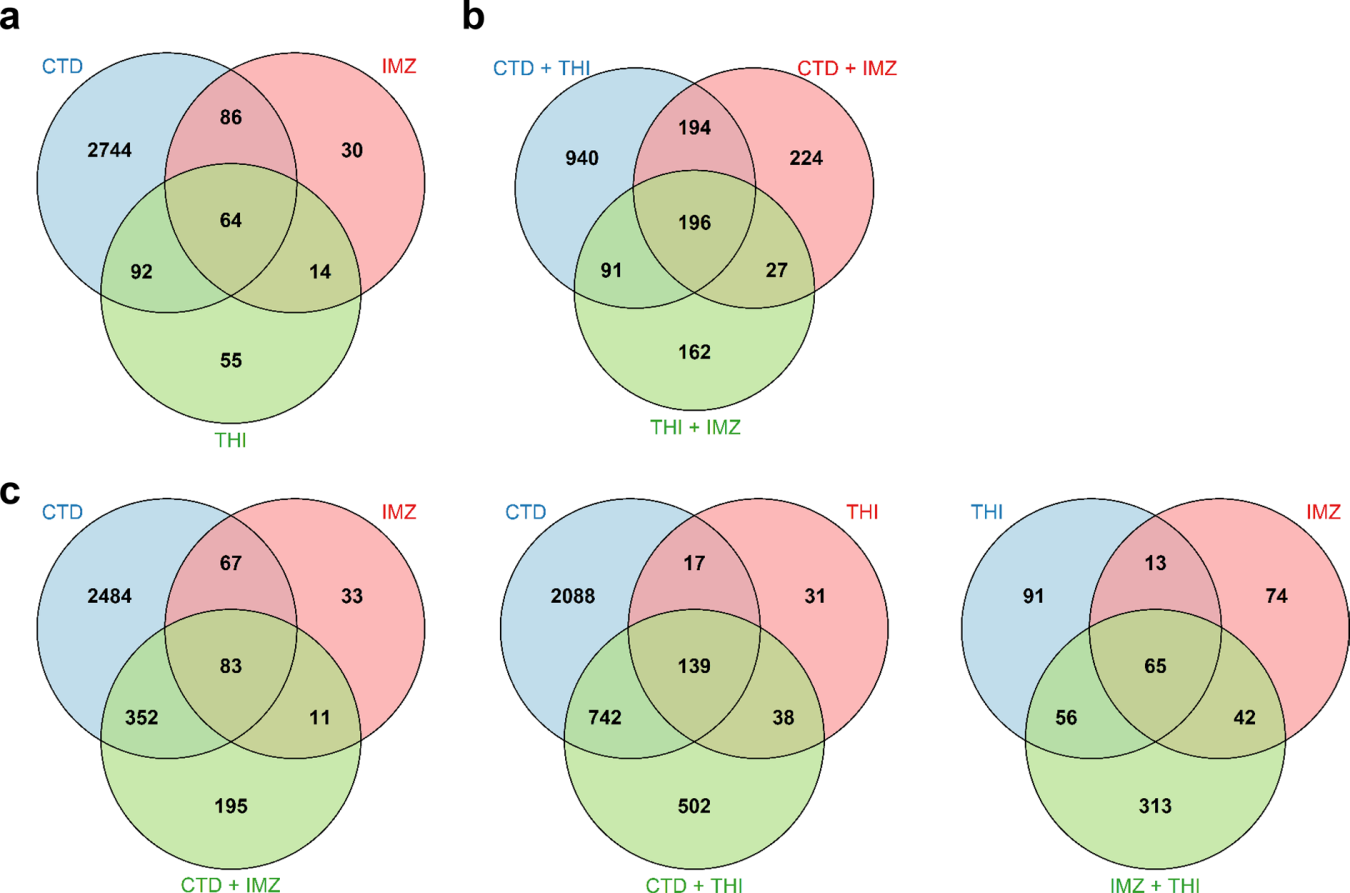
Venn diagrams showing the overlaps and differences in the number of genes regulated by single compounds (**a**), mixtures (**b**) and single compounds and their corresponding mixtures (**c**) in rat liver. Only genes with a *q*-value < 0.05 were included

In a next step, we analyzed the overlaps between sets of DEGs and visualized them in Venn diagrams. In single treatment condition, a total of 64 common genes were differentially regulated by IMZ, THI and CTD (Fig. 3a). GO term analysis revealed an enrichment of *xenobiotic metabolic process* (GO:0006805) and similar ones for common DEGs (Supplementary Figure S1). 156 (92 + 64) genes were commonly affected by CTD and THI, while 150 (86 + 64) genes were commonly affected by CTD and IMZ. IMZ and THI shared 78 (64 + 14) differentially regulated genes. Among these shared DEG sets, we identified GO terms related to *response to glucocorticoid* and *DNA packaging* (GO:00513844, GO:0006323) as well as *steroid and fatty acid metabolic process* (GO:0008202, GO:0006631) (Supplementary Table S4). 92% of the genes regulated by CTD were exclusively regulated by this compound and not affected by any of the other compounds. This figure was 15% and 24% for IMZ and THI, respectively. For the genes exclusively regulated by CTD, we observed strong enrichment of GO terms related to *cell division* and *DNA replication* (GO:0051301, GO:0006260).

In mixture treatment condition, a total of 196 common genes were differentially regulated by the 3 different combinations (Fig. 3b). 390 (196 + 194) genes were commonly affected by CTD + THI and CTD + IMZ while 287 (196 + 91) genes were commonly affected by CTD + THI and THI + IMZ. CTD + IMZ and THI + IMZ shared 223 (196 + 27) differentially regulated genes.

Comparison between CTD, IMZ and their mixture showed that the number of new genes found solely in mixture treatment condition (i.e., that were not regulated in single condition by either CTD or IMZ) is 195, representing 30% (195/641) of total regulated genes (Fig. 3c). 352 genes in the mixture treatment condition were regulated solely by CTD (~ 55%). A similar trend is observed for mixture THI + CTD with 52% of genes being regulated solely by CTD (742 out of 1421) while around 35% of new genes were regulated (502 out of 1421). On the contrary, for THI + IMZ the proportion of new genes regulated solely in mixture is considerably higher, reaching 66% (313/476). 56 genes in mixture treatment condition are genes that were regulated solely by THI (~ 12%) while 42 genes were solely regulated by IMZ (~ 9%).

To confirm the data acquired by RNA-Seq, the expression of selected genes was verified using qRT-PCR. In addition, RNA samples from the remaining dose levels of treatment with individual compounds or mixtures were used to quantify the full dose–response curve of the selected transcripts. We decided to choose genes that were among the most differentially regulated (both up and down) and which feature a biological interest (Table 2). Supplementary Figure S3 presents volcano plots for the different treatment conditions, highlighting the top ten differentially regulated genes. Xenobiotic-metabolizing enzyme (XME)-coding genes (i.e*., Aldh1a1*, *Cyp2b2*, and *Cyp3a23/3a1*) represent almost one-third of the selected ten genes. Genes involved in bile acid synthesis (*Cyp7a1*) and uptake/disposition (*Slc6a1* and *Abcc3*) were also among the top differentially regulated genes. Surprisingly, the other genes among the most regulated ones imply neuronal functions and neurotransmitter/hormone pathway genes (*Ky* and *Mme*).

**Table 2:** Top ten differentially regulated genes (fold change; *q* < 0.05) after treatment with compounds alone and in mixtures

	Gene	Function	THI	IMZ	CTD	THI + IMZ	THI + CTD	IMZ + CTD
Up-regulation	*Abcc3*	Transporter	3.67	3.28	2.94	3.88	3.95	2.37
	*Aldh1a1*	Enzyme	2.77	2.31	2.55	2.75	3.14	2.09
	*Cyp2b2*	Enzyme	3.29	2.82	2.82	3.11	3.45	2.01
	*Cyp3a23/3a1*	Enzyme	2.60	2.82	1.59	2.41	2.47	1.78
	*Mme*	Enzyme	2.97	2.59	3.43	2.46	4.35	2.70
Down-regulation	*Cyp7a1*	Enzyme	− 1.80	− 1.18*	− 1.51	− 1.89	− 1.50	− 1.55
	*Eln*	Other	− 1.97	− 2.08	− 1.80	− 2.04	− 1.91	− 1.79
	*Ky*	Enzyme	− 2.41	− 1.65	− 3.21	− 2.32	− 2.89	− 1.65
	*Megf11*	Other	− 2.37	− 2.14	− 3.18	− 2.26	− 2.87	− 2.87
	*Slc6a1*	Transporter	− 1.77	− 1.37*	− 1.96	− 1.80	− 1.93	− 1.63

The asterisk indicates non-significant fold change

Direction of gene regulation correlated well for all genes (except *Ky*) between RNA-Seq and qRT-PCR analyses (Supplementary Figure S4). Quantitatively, *Cyp3a23/3a1* and Mme up-regulations were underestimated by RNA-Seq. *Abcc3* and *Cyp3a23/3a1* showed a dose-dependent up-regulation for all treatment groups. For the genes that were positively regulated, the magnitude of up-regulation increased with rising doses.

### Ingenuity pathway analysis

#### Upstream effectors

**Fig. 4:**
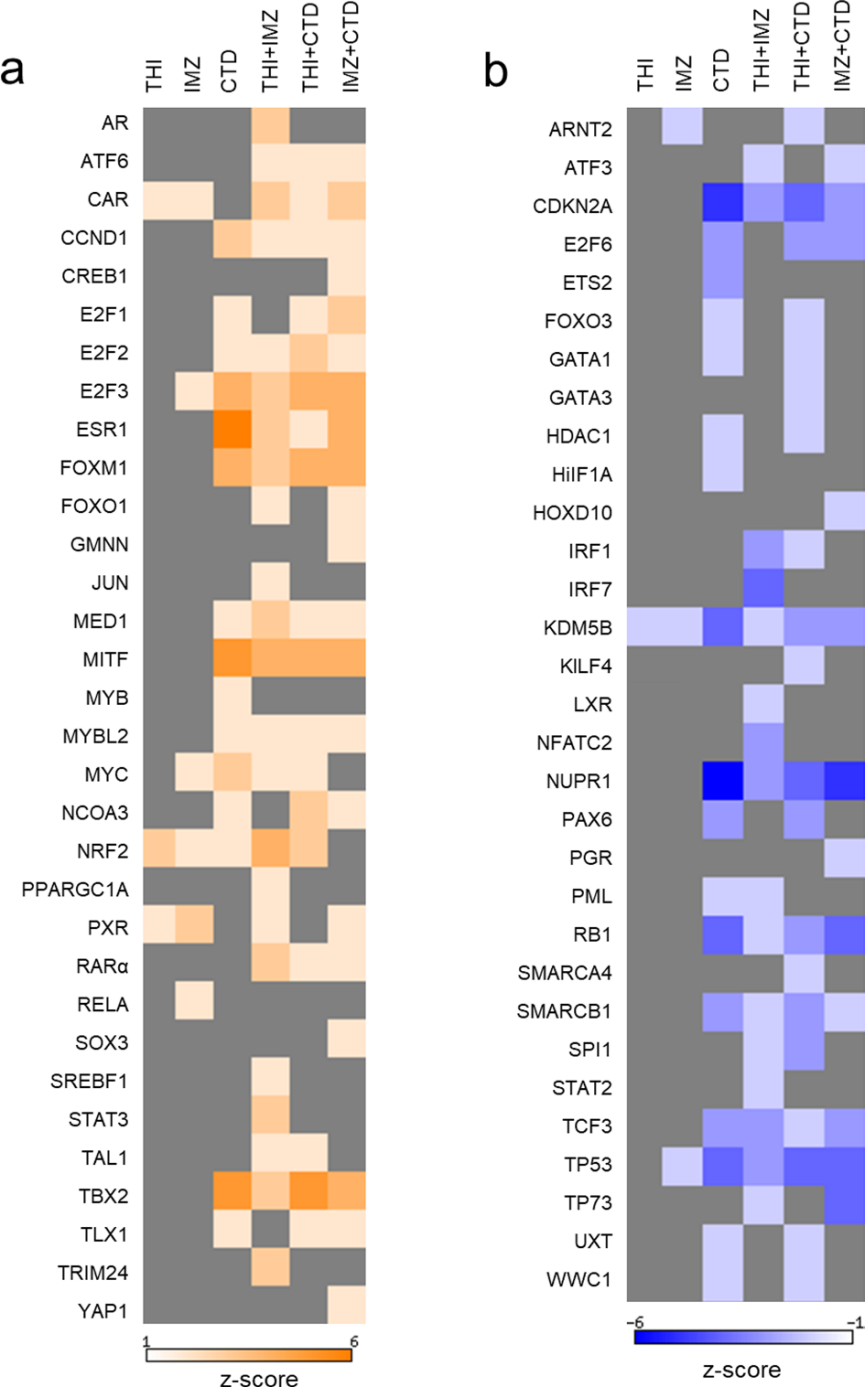
Transcription regulators predicted to be affected by THI, IMZ, CTD and their mixtures in rat livers. The list of genes significantly regulated by pesticides in rat hepatocytes as revealed by RNA-Seq (*q* < 0.05) was comparatively analyzed using Ingenuity Pathway Analysis. Upstream regulator analysis was performed and the *p* value (calculated by Fisher’s exact test right-tailed) was used to identify transcription regulators significantly associated with genes regulated by pesticides. The *p* value indicates the probability of association of a set of genes in the dataset with a transcription regulator by random chance alone. A *p* value < 0.05 was considered significant. The IPA activation *z*-score was used to predict transcription regulators that are increased or decreased by pesticides. Only transcription regulators with a *z*-score ≥ 2, predicting a significant increase (**a**), or a z-score ≤ − 2, predicting a significant decrease (**b**), were considered. Missing values (i.e., − 2 < *z*-score < 2) are depicted in gray

We performed an upstream analysis to predict the possible activation/inhibition of transcription regulators involved in the toxicological response to the pesticides. As shown in Fig. 4, many transcription regulators were predicted to be affected in either activation or inhibition direction. Using the single compound treatment datasets, the highest number of predicted regulations was observed for CTD, while THI and IMZ modulated comparatively few transcription regulators. This is in line with the differences in the overall numbers of regulated genes.

THI and IMZ showed a similar profile in the activation of transcription regulators with increase of CAR, PXR and NRF2 (Fig. 4a). CAR and PXR are nuclear receptors involved in xenobiotic/endobiotic metabolism, while NFR2 is a transcription factor involved in response to oxidative stress. CTD was also predicted to activate NFR2 along with ESR1 (estrogen receptor) and many regulators involved in cell cycle and cell proliferation (E2F1-3, FOXM1, MYB, MYBL2 and MYC). In mixture (i.e., with THI or IMZ), a similar pattern of activation was observed. Interestingly, the mixture of THI and IMZ appeared to activate much more transcription regulators than single treatment, and featured a pattern of activation similar to those of THI + CTD and IMZ + CTD.

The only transcription regulator predicted to be inhibited after THI treatment was KDM5B (Fig. 4b), while IMZ also inhibited ARNT2 and TP53. CTD inhibited the highest number of regulators with strongest decreases in the tumor suppression-related proteins CDKN2A, KDM5B, RB1 and in multi-functional protein TP53. THI + CTD and IMZ + CTD featured a similar pattern of inhibition as observed for CTD alone. The mixture of THI and IMZ inhibited much more transcription regulators than single treatment, and features a pattern of inhibition similar to IMZ + CTD.

### Pathways

**Fig. 5:**
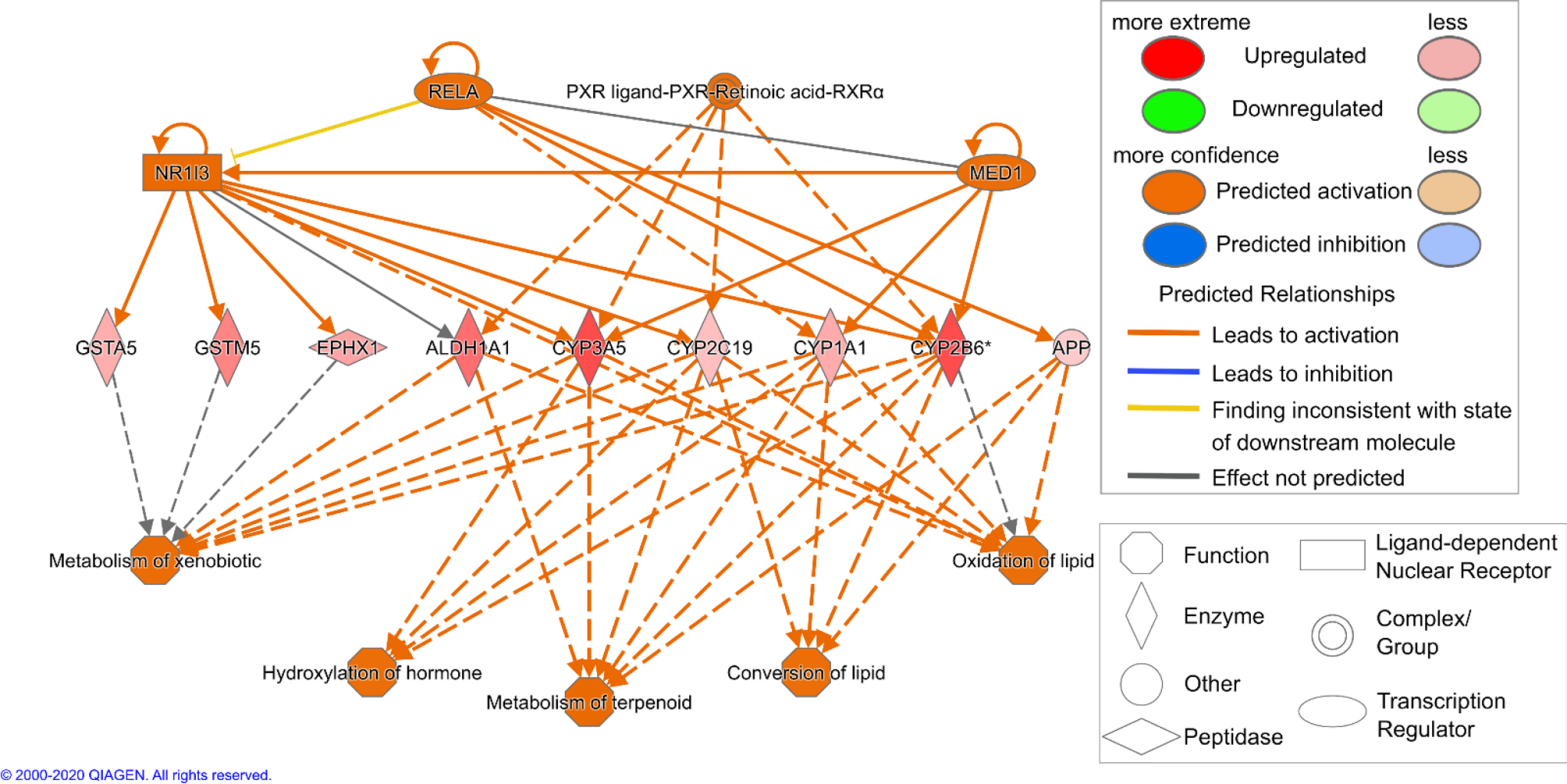
Network of transcriptional NR1I3-mediated response elicited by pesticides (here shown: IMZ) in rat liver, as computed with Ingenuity Pathway Analysis software. Continuous lines indicate direct functional relationships, dashed lines indicate indirect relationships (i.e., do not require physical contact between the two molecules, as defined by IPA). Network nodes are labeled with the gene name and the functional classification of genes is denoted by their different shapes. The human genes *CYP2B6, CYP2C19, CYP3A5, GSTA5 and GSTM5* correspond to the rat genes *Cyp2b2, Cyp2b6, Cyp3a23/3a1, Gsta2*, and *Gstm1*, respectively

Networks consist in functionally related transcripts and proteins involved in a biological response connected with predictions of adverse outcomes at the tissue and organ level, as well as with predictions of potential regulators involved in the changes seen at the mRNA and protein levels. Considering the similar spectrum of reported effects by all three pesticides, we hypothesized that a common network should exist between the three pesticides, aside from possible other compound-specific networks. Therefore, we used the subgroup of 64 genes that were regulated by all 3 pesticides (see corresponding Venn diagram in Fig. 3a). Figure 5 depicts the network of NR1I3 (i.e., CAR) and PXR. The transcription regulators NR1I3 and MED-1 are interconnected and regulate different enzymes such as ALDH1A1, CYP3A5, CYP2B6, EPHX1, GSTA5 and GSTM5, which in turn activate the functions *metabolism of xenobiotic*, *metabolism of terpenoid*, *conversion and oxidation of lipid*.

In a second step, we investigated whether the specific subgroups of each pesticide would retrieve any networks. No networks were predicted for IMZ and THI. This is in line with only very few enriched GO terms (Supplementary Figure S1) for both pesticides. For CTD, a network leading to the function *DNA repair* is depicted (Supplementary Figure S5). In this network, BRCA1 plays a key role by regulating downstream targets involved in gene expression (IFNG), checkpoint regulation (CHK1, PLK1) or by being part of complexes A, B, and C which are involved in homologous recombination. Important actors in the response to DNA double-strand breaks (i.e., ATR, MDC1, MCPH1 and H2AX) are also clearly up-regulated by CTD treatment.

### Evaluation of mixture effects

Characterization of mixture effects relies on the use of mathematical models, each model having different features (Foucquier and Guedj 2015; Lasch et al. 2020). In the context of this study, proper characterization of mixture effects towards gene regulation will be described in Sect. 3.3.1. Additionally, we considered it worth to describe relevant observations that may indicate mixture effects.

### Top ten differentially regulated genes

**Fig. 6:**
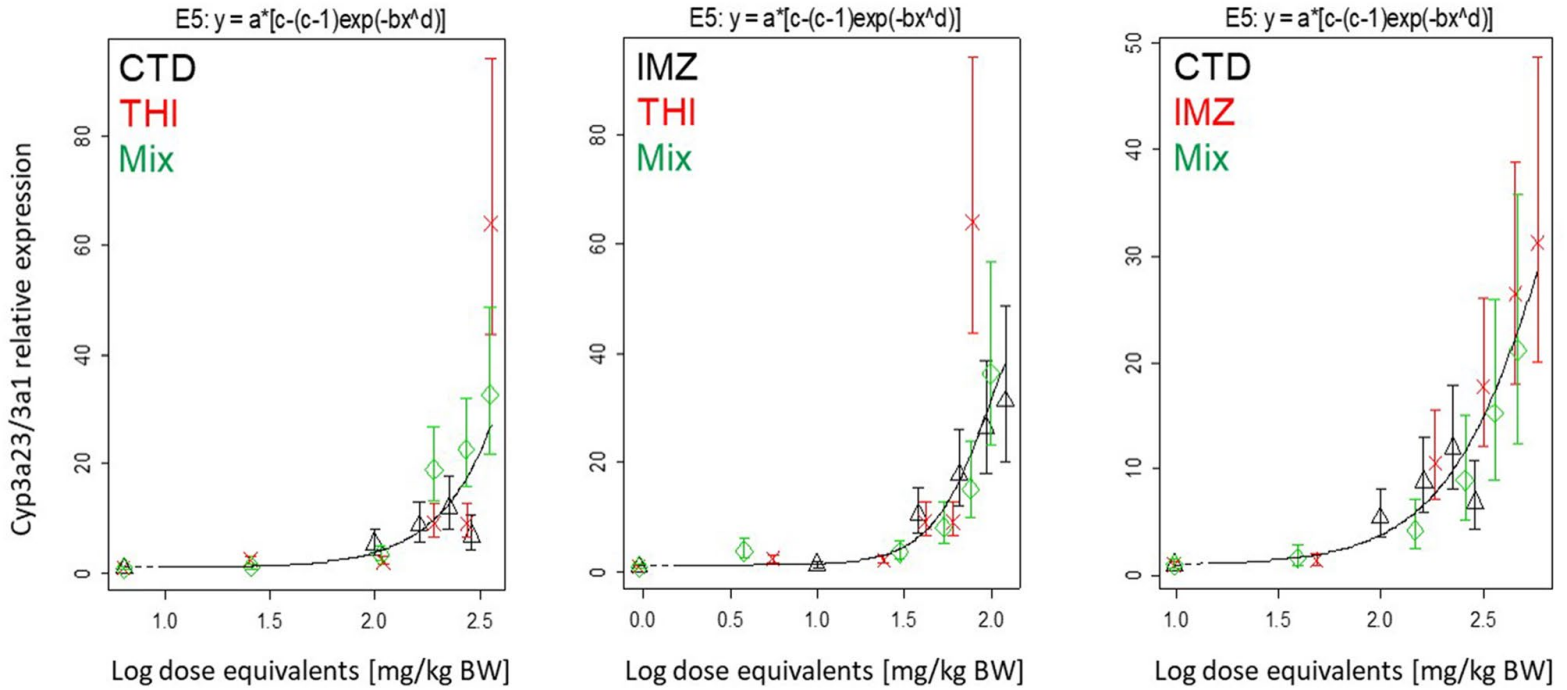
Representative dose–response modeling of gene expression *Cyp3a23/3a1*. The curves represent the four-parameter exponential model. For testing of mixture effects, the dose–response data for the single compounds and mixtures were compared using the benchmark (BMD) dose modeling software PROAST. Data are shown as means and SD. The concentration–response of the mixture (green diamonds) shows no derivation from the overall concentration–response fit, indicating that dose addition can be assumed. Data for gene regulation can be found in Supplementary Table S7

To assess mixture effects, the dose–response data obtained by qRT-PCR was analyzed via PROAST. Genes showing no regulation or dissimilar gene regulation direction between single treatment and mixture were not suitable for this analysis. Therefore only *Abcc3*, *Aldh1a1*, *Cyp3a23/3a1* and *Mme* were analyzed. The data points for all three mixtures fit the curve derived from the respective single compounds (one representative modeling is shown for *Cyp3a23/3a1* in Fig. 6, modeling for other genes can be found in Supplementary Figure S6). According to Kienhuis et al. (2015), this indicates dose addition for the combination of the two respective substances.

### Cluster analysis

**Fig. 7:**
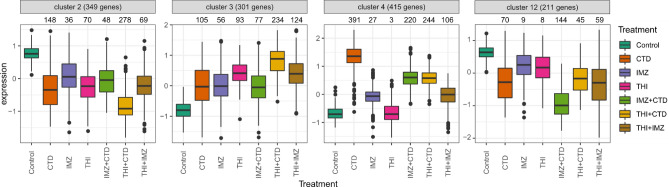
Boxplot of scaled gene expression per treatment per cluster. All 3837 DGEs were grouped by kmeans clustering into 12 clusters among which 4 clusters are presented here exemplarily. The numbers above each boxplot indicate the number of significant DGEs per treatment and cluster

To gain insight into regulated pathways and associated biological pathways among all DEGs (*n* = 3837), we performed kmeans clustering to group co-regulated genes into 12 different clusters (Supplementary Figure S7). Cluster 2 and 3 reflect down- or up-regulation compared to control across all treatments, respectively (Fig. 7). We tested for GO term enrichment using hypergeometric test per cluster and summarized the results in a heatmap (Supplementary Figure S8, Supplementary Table S6). Cluster 2 contains mainly down-regulated genes (e.g., *Tlr7*, *Sema4a*) enriched for *adaptive immune response, response to virus* and *T cell activation*. The up-regulated genes of cluster 3 are related to enriched GO terms, like *xenobiotic metabolic process* and *lipid modification* that are reflected by the network shown in Fig. 5. Cluster 4 illustrates a gene expression pattern where mostly CTD and its mixtures induce up-regulation of genes related to *DNA replication, cell division* and *cell cycle*. The genes of cluster 12 are mainly down-regulated by IMZ + CTD treatment and enriched for GO terms, such as *rhythmic process* and *response to virus.* Most of the remaining clusters (6–11) are dominated by CTD-induced gene regulation (Figure S6).

In the following subsections, observations in regards to hypothetical mixture effects are described.

### THI + IMZ

For the mixture THI + IMZ, Venn diagram analysis revealed 313 genes differentially regulated solely in the mixture condition (ca. 66% of total differentially regulated genes, see Fig. 3c). 185 of those genes are retrieved in the clusters 3, 4 and 12 (53, 81, and 51, respectively). Among the 313 specifically differentially regulated genes, we found GO terms enriched such as *negative regulation of innate immune response* (GO:0045824, mainly cluster 2) and *cell division* (GO:0051301, mainly cluster 4) (Supplementary Figure S2).

### IMZ + CTD

**Fig. 8:**
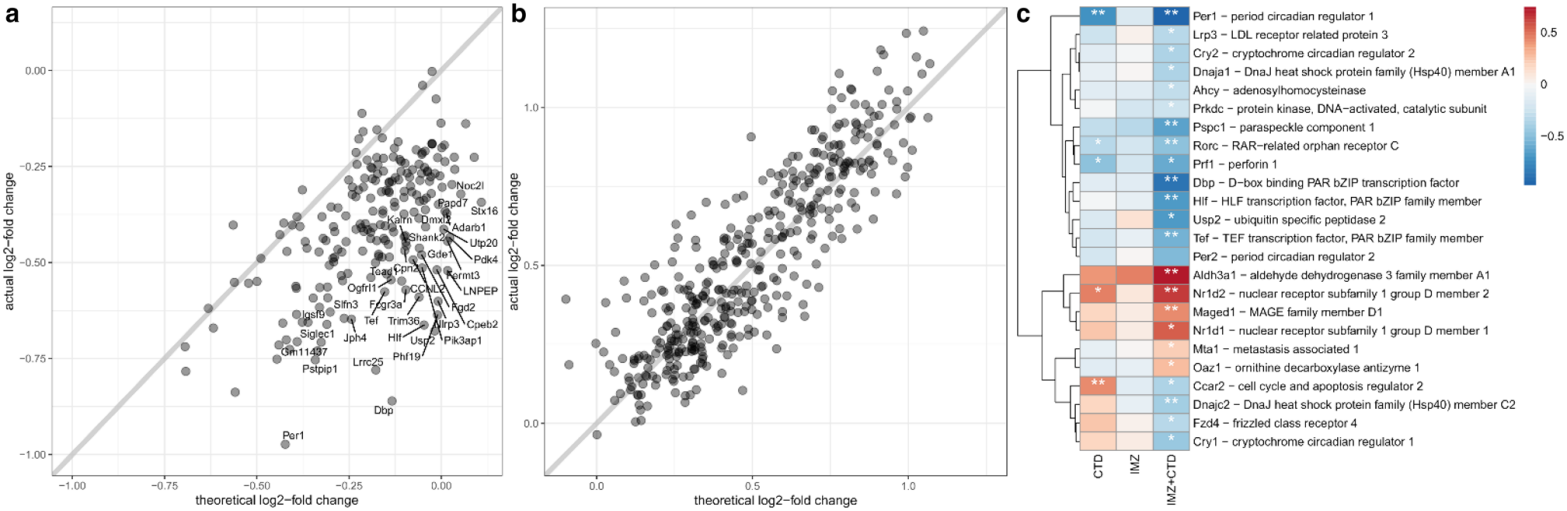
Gene expression changes specific for IMZ + CTD. Scatterplot of actual versus theoretical log_2_-fold change for genes from cluster 12 (**a**) and cluster 4 (**b**). Grey line indicates the theoretical additive response of IMZ + CTD. Data for all clusters can be found in Supplementary Figures S9 to S11. Heatmap of log_2_-fold change for genes related to circadian rhythm differentially regulated by IMZ + CTD mixture (**c**)

For the mixture IMZ + CTD, Venn diagram analysis revealed 195 genes differentially regulated solely in the mixture condition (ca. 30% of total differentially regulated genes, see Fig. 3c). 124 of those genes are retrieved in the clusters 3 and 12 (29 and 95, respectively). It is noteworthy that the genes of cluster 12 show the most pronounced down-regulation by IMZ + CTD treatment compared to treatment by IMZ or CTD. Figure 8a shows that the actual log_2_-fold change is stronger than the theoretical one for numerous genes suggesting synergistic interactions for this gene set (in contrast to cluster 4, Fig. 8b). GO enrichment analysis for the set of 195 specifically differentially regulated genes revealed *regulation of circadian rhythm* (GO:0042752) including many key regulators like, Cry1, Cry2, Dbp, Nr1d2 and Per1 (Fig. 8c).

### THI + CTD

For the mixture THI + CTD, Venn diagram analysis revealed 502 genes differentially regulated solely in the mixture condition (ca. 35% of total differentially regulated genes, see Fig. 3c). However, GO enrichment analysis for these genes reveals only slight hints for genes related to *lipid homeostasis* (Supplementary Figure S2). 462 of those genes are retrieved in the clusters 1, 2, 3 and 5 (100, 151, 125, and 86, respectively). In cluster 1–5, we observed a strong mixture effect, for cluster 2 and 3 even more pronounced than for the single treatments with CTD or THI (Supplementary Figures S7 and S8). This might point to a synergistic effect for some genes of these clusters.

## Discussion

In a previous paper, we published the results of a 28-day repeated-dose oral exposure study with the three pesticides IMZ, THI and CTD in rats: the outcomes included liver weight increase, hepatocellular hypertrophy, cytoplasmic degeneration and toxicokinetic alterations (Alarcan et al. 2020). In the present study, the goal was to investigate the associated hepatic molecular MoA of the pesticides IMZ, THI and CTD, alone or in mixtures.

RNA-Seq analysis revealed a high number of differentially regulated genes by CTD, while THI and IMZ modified the gene expression more moderately. In detail, CTD differentially regulated 13 and 15 times more genes than THI and IMZ, respectively. This much stronger impact on gene expression could either reflect a higher potency of CTD or a different and/or additional MoA which does not necessarily become apparent at the histopathological level due to the short treatment period. Despite the relatively small subset of commonly DEG (i.e., 64 genes), we were able to describe a consistent network depicting the transcriptional CAR and PXR-mediated response leading to the activation of metabolism-associated functions (metabolism of xenobiotic, metabolism of terpenoid, conversion and oxidation of lipid). Since all three pesticides induced comparable effects on the liver (increase in organ weight, hepatocellular hypertrophy, decrease in triglyceride content) and with a similar magnitude, it is coherent to find a common molecular MoA between the three substances. The common network includes ALDH1A1, CYP2B6 and CYP3A5 (the orthologs of rat Cyp2b2 and rat Cyp3a23/3a1, respectively), whose gene expressions were among the top up-regulated ones detected by RNA-Seq and confirmed by qRT-PCR. These three XME are respectively located in the mitochondria and in the endoplasmic reticulum of the cells. This is in line with the observation of liver hypertrophy following exposure to IMZ, THI and CTD, as the nuclear receptor-mediated induction of metabolic enzymes causes an increase in endoplasmic reticulum, peroxisomes and/or mitochondria. Previous studies showed that IMZ activates both human and mouse PXR and also human CAR (Lichtenstein et al. 2020; Yoshimaru et al. 2018). Besides, THI activated human PXR and PPARγ while CTD showed no effect on human PXR or CAR (Lichtenstein et al. 2020). Predictions by IPA on transcription regulators further support this idea, as CAR and PXR, two master regulators of XME, were activated by THI and IMZ. However, in the case of CTD, none of those nuclear receptors was predicted for activation. Instead, the strongest activation was predicted for the estrogen receptor (ER). Nonetheless, since CTD is not considered an agonist of ER (according to the ToxCast database), this hypothesis might not be valid in the present case. Besides, particular caution needs to be taken when considering ligand properties towards xenobiotic metabolism-associated nuclear receptors as multiple inter-species differences are documented (Omiecinski et al. 2011; Timsit and Negishi 2007; Wang et al. 2012).

In a second step, we aimed at identifying specific networks associated with the unique gene expression changes for each pesticide. A DNA repair network was predicted for CTD, possibly indicating a genotoxic potential. Our present data do not allow concluding on such toxicological property, but it is noteworthy that JMPR Meeting reported three in vitro studies where CTD was found positive in gene mutation and chromosomal aberration assays (JMPR 2010). Those three studies indicated a clastogenic potential, which is in line with the different regulators included in the network predicted by IPA, such as BRCA1, ATR, MDC1, MCPH1 and H2AX (Bonner et al. 2008; Hustedt and Durocher 2016). Interestingly, a study on pyrrolizidine alkaloids reported the induction of a DNA damage response involving the up-regulation of *Rad51* and *Ddias* that were also induced our study (Ebmeyer et al. 2020). For THI or IMZ, no specific networks or evident GO enrichment patterns were observed.

When doing mixture toxicity experiments, it is necessary to evaluate the data by means of one or several mathematical models to come to conclusions on the behavior of compounds in mixture (Foucquier and Guedj 2015; Lasch et al. 2020; Zhao et al. 2010). A prerequisite in mixture analysis involves the determination of the similarity or dissimilarity in the mode of action of the compounds of study. This determines the choice of mathematical model to use (Cedergreen et al. 2008; EFSA 2013). Furthermore, it is necessary to have quantitative data from multiple concentration/dose levels for a proper use of the mathematical models. In the case of transcriptomics analysis, each investigated gene should be technically considered as a single endpoint, so that two compounds in combination are unlikely to show the exact same type of mixture effect for every gene. As a result, the strict denomination for a mixture appears elusive. Besides, evaluation of mixture effects with recommended mathematical models is not feasible if based on solely one dose level. However, PCA could be seen as a multivariate technique for MoA evaluation. PCA is a statistical method permitting the visualization of high-dimensional data by new sets of variables called principal components, thereby enabling the identification of sample clusters (Ringner 2008; Todorov et al. 2018). In our study, single reference compounds and their mixtures (especially THI + IMZ) showed relative overlaps of their clusters, meaning that they overall show similar gene expression signature profiles according to PC1 and PC2. Dose addition would be then legitimately considered. In case of THI + CTD, the samples were not overlapping with THI or CTD indicating potential independent modes of action. Similarly, PCA revealed hidden structures in gene expression data of metal mixtures reflecting unique transcriptional signatures and suggesting interaction among the single compounds (Tilton et al. 2011; Vandenbrouck et al. 2009). Moreover, PC1 and PC2 account together only for up to 30% of the transcriptomics variation so that the remaining variance might reveal further separation of mixtures and the single compound defined as reference hinting towards non-additive effects.

Besides this multivariate approach to determine quantitative mixture effects for gene clusters, we also applied a qualitative assessment using Venn diagrams followed by GO term enrichment analysis of specific gene sets. Our results showed that for each mixture a significant proportion of new genes (i.e., not regulated by treatment with single compounds) was regulated (from 30 up to 66%). For some combinations, gene enrichment analysis revealed enrichment of GO terms for those specific subsets of genes, such as circadian rhythm. It has been shown that ~ 10% of the rat liver transcriptome are rhythmically expressed (Storch et al. 2002) including genes related to xenobiotic detoxification (Tahara and Shibata 2016). The treatment with IMZ + CTD resulted in pronounced inhibition of clock-controlled genes *Dbp*, *Tef*, and *Hlf* which regulate expression of genes encoding detoxification enzymes. Possibly this mixture disrupted the circadian rhythm of expression of certain genes, as indicated by altered gene expression levels, and resembling the effects of 4-OH-CB107 on the rat liver transcriptome (Ochiai et al. 2018).

Therefore, although being unable to characterize it with a mathematical model, it seems that mixture effects take place. Thus, a transcriptomics approach (or any other omics technology) may bring substantial benefit in the framework of chemical mixtures as they permit to uncover the whole spectrum of biological effects of substances and could reveal as shown here for IMZ + CTD some particular effects that would be laborious to reveal using traditional single target apical endpoints. A two-step investigation (untargeted transcriptomics approach followed by endpoint confirmation using a targeted technique) could be an efficient strategy to be applied in the context of chemical mixtures.

The quantitative and qualitative approaches we applied to assess mixture effects on transcript responses resulted in common and specific gene expression patterns. While for specific gene expression patterns different MoA and thus independent action may be assumed, common patterns point at a common MoA and at dose addition. For a limited set of commonly affected genes that were studied by qRT-PCR, we indeed observed additive effects: irrespective of the combination, dose addition was described for *Aldh1a1* or *Cyp3a23/3a1*. As discussed previously, those genes are suspected to reflect the main biological effect of hypertrophy. Thus, it is of note that the same concordance in the type of mixture effect was described in vivo over three different layers of biological organization, i.e., molecular (genes), cellular (hepatocellular hypertrophy), and tissue (increase in liver weight).

## Conclusions

In the present study, we comparatively analyzed transcriptome expression in rat hepatocytes after 28-day exposure to IMZ, THI and CTD alone or in mixtures. RNA-Seq revealed drastic regulation of gene expression by CTD, while IMZ and THI moderately affected gene expression. Furthermore, we suggest that pesticide-induced hepatotoxicity is associated with nuclear receptor activation as the most differentially regulated genes involve some key phase I XME and transport proteins. The data presented in the current study provide new insights into the molecular mechanism of hepatotoxicity of IMZ, THI and CTD. While bioinformatics analysis revealed some evidence for possible non-additive regulation of certain groups of transcripts by the mixtures, results from the evaluation of individual top-regulated genes followed the additivity concept.

## Supplementary Information

Below is the link to the electronic supplementary material.

Supplementary file1 (DOCX 4777 KB)

## Data Availability

The raw RNA sequencing data are available from GEO under accession number GSE153986.

## Abbreviations

AOP: Adverse outcome pathway
BMD: Benchmark dose
CTD: Clothianidin
CAG: Cumulative assessment group
CAR: Constitutive androstane receptor
CYP: Cytochrome P450
DAR: Draft assessment report
EFSA: European Food Safety Authority
IMZ: Imazalil
IPA: Ingenuity pathway analysis
MoA: Mode of action
NOAEL: No-Observed-Adverse-Effect-Level
OECD: Organization for Economic Co-operation and Development
PPARα/γ: Peroxisome proliferator-activated receptor α/γ
PXR: Pregnane X receptor
RNA-Seq: Total RNA sequencing
RPF: Relative potency factor
THI: Thiacloprid
XME: Xenobiotic-metabolizing enzyme
