# Supplementary material for: Transcriptomics analysis of hepatotoxicity induced by the pesticides imazalil, thiacloprid and clothianidin alone or in binary mixtures in a 28-day study in female Wistar rats
Source: Arch Toxicol. 2021 Jan 11;95(3):1039–53. doi: 10.1007/s00204-020-02969-y (PMC7904562; doi:10.1007/s00204-020-02969-y)
Supplement: Supplementary file 1 — Supplementary file1 (DOCX 4777 KB) [file 204_2020_2969_MOESM1_ESM.docx]

**Supplementary results section**

**Transcriptomic analysis of hepatotoxicity induced by pesticides imazalil, thiacloprid and clothianidin alone or in binary mixtures in a 28-day study in female Wistar rats**

Jimmy Alarcan^a#^, Heike Sprenger^a#^, Julia Waizenegger^a#^, Dajana Lichtenstein^a^, Claudia Luckert^a^, Philip Marx-Stoelting^b^, Alfonso Lampen^a^, Albert Braeuning^a*^

^a^ German Federal Institute for Risk Assessment, Department of Food Safety, Max-Dohrn-Straße 8-10, 10589 Berlin, Germany

^b^ German Federal Institute for Risk Assessment, Department Pesticides Safety, Max-Dohrn-Straße 8-10, 10589 Berlin, Germany

***** Correspondence: Albert.Braeuning@bfr.bund.de; Tel.: +49 (30) 18412-25100; Adress: German Federal Institute for Risk Assessment, Department of Food Safety, Max-Dohrn-Straße 8-10, 10589 Berlin, Germany.

^#^ Equally contributed to the publication

**Table S1** Summary of the RNA-Sequencing read data and 31 rat liver samples treated by pesticides. Raw reads were mapped to the reference genome Rnor_5.0 and uniquely mapped reads were used for further analyses

| Sample ID | Treatment | Dose | Number of raw reads (million) | Number of mapped reads (million) | Percentage of mapped reads | Number of uniquely mapped reads (million) | Percentage of uniquely mapped reads |
| --- | --- | --- | --- | --- | --- | --- | --- |
| 1 | control | - | 159.706 | 138.859 | 86.95 | 101.23 | 72.90 |
| 2 | control | - | 50.549 | 43.491 | 86.04 | 32.726 | 75.25 |
| 3 | control | - | 69.434 | 57.921 | 83.42 | 42.804 | 73.90 |
| 4 | control | - | 100.756 | 86.347 | 85.70 | 65.71 | 76.10 |
| 5 | control | - | 140.004 | 119.418 | 85.30 | 88.212 | 73.87 |
| 6 | control | - | 101.619 | 87.82 | 86.42 | 66.91 | 76.19 |
| 7 | control | - | 88.824 | 76.144 | 85.72 | 55.241 | 72.55 |
| 8 | control | - | 57.984 | 51.262 | 88.41 | 38.51 | 75.12 |
| 9 | Thiacloprid | high | 54.303 | 47.093 | 86.72 | 35.335 | 75.03 |
| 10 | Thiacloprid | high | 83.849 | 72.797 | 86.82 | 54.792 | 75.27 |
| 11 | Thiacloprid | high | 63.369 | 53.319 | 84.14 | 39.059 | 73.26 |
| 12 | Thiacloprid | high | 67.777 | 56.051 | 82.70 | 42.278 | 75.43 |
| 13 | Imazalil | high | 72.857 | 63.135 | 86.66 | 46.327 | 73.38 |
| 14 | Imazalil | high | 66.116 | 55.094 | 83.33 | 40.217 | 73.00 |
| 15 | Imazalil | high | 61.593 | 53.419 | 86.73 | 40.989 | 76.73 |
| 16 | Imazalil | high | 70.718 | 60.156 | 85.06 | 44.064 | 73.25 |
| 17 | Clothianidin | high | 56.079 | 48.362 | 86.24 | 35.414 | 73.23 |
| 18 | Clothianidin | high | 68.624 | 57.514 | 83.81 | 38.436 | 66.83 |
| 19 | Clothianidin | high | 52.161 | 45.881 | 87.96 | 31.82 | 69.35 |
| 20 | Thiacloprid+Imazalil | high | 64.498 | 56.355 | 87.37 | 42.283 | 75.03 |
| 21 | Thiacloprid+Imazalil | high | 60.017 | 51.076 | 85.10 | 40.044 | 78.40 |
| 22 | Thiacloprid+Imazalil | high | 62.138 | 54.344 | 87.46 | 40.823 | 75.12 |
| 23 | Thiacloprid+Imazalil | high | 69.97 | 60.195 | 86.03 | 45.706 | 75.93 |
| 24 | Thiacloprid+Clothianidin | high | 60.824 | 52.641 | 86.55 | 41.016 | 77.92 |
| 25 | Thiacloprid+Clothianidin | high | 69.258 | 57.453 | 82.96 | 44.545 | 77.53 |
| 26 | Thiacloprid+Clothianidin | high | 68.497 | 59.063 | 86.23 | 47.096 | 79.74 |
| 27 | Thiacloprid+Clothianidin | high | 54.595 | 46.353 | 84.90 | 35.783 | 77.20 |
| 28 | Imazalil+Clothianidin | high | 50.684 | 41.723 | 82.32 | 31.92 | 76.50 |
| 29 | Imazalil+Clothianidin | high | 53.764 | 46.029 | 85.61 | 36.627 | 79.57 |
| 30 | Imazalil+Clothianidin | high | 53.595 | 45.72 | 85.31 | 35.998 | 78.74 |
| 31 | Imazalil+Clothianidin | high | 51.951 | 42.774 | 82.34 | 32.94 | 77.01 |

**Table S2** Summary of primers used for q-PCR analysis

| Gene | Sequence (5' to 3') | |
| --- | --- | --- |
| Abcc3 | F: | AGATCGCAGAGACAGGCAAT |
|  | R: | CCAGCATACAGGAGGCAGAT |
| Aldh1a1 | F: | AAGAAGGGGACAAGGCAGAT |
|  | R: | GCGTCCTCTCTCTGAAGCAT |
| β-actin | F: | CGTCCACCGCAAATGCTT |
|  | R: | GTTTTCTGCGCAAGTTAGGTTTTGT |
| Cyp3a23/3a1 | F: | CTGACAGACAAGCAGGGATG |
|  | R: | TGGGTTCCAAGTCGGTAGAG |
| Cyp7a1 | F: | CTCGCTATTCTCTGGGCATC |
|  | R: | GAGGCTGCTTTCATTGCTTC |
| Eln | F: | TGCTACTGCTTGGTGGAGAA |
|  | R: | TCCTTGTCCTGTGGGTTTTC |
| Ky | F: | CCTGGTCTCCCTCCACTACA |
|  | R: | TCAGCAGTTGCCAGTTTTTG |
| Mme | F: | AGCCTCAGCCGAAACTACAA |
|  | R: | GCATTCTCCATGTTCCCATT |
| Slc6a1 | F: | TCGTCATCTTCTCCATCGTG |
|  | R: | GGTATGCCAAGAATGCCAGT |

For Tables S3 to S6, please refer to the Excel file for visualization of the data.

**Table S3** Results of differential gene expression analysis for all treatments versus control conditions. Significant results (q-value < 0.05) are highlighted in green and log_2_-fold changes are color coded (red for positive and blue for negative values)

**Tables S4** Comparison of GO enrichment of DEG sets for single compound treatments. Table shows all GO terms with significant enrichment (adjusted p-value < 0.05) across specific or non-specific DEG sets. Color indicates –log_10_(p-value)

**Table S5** Comparison of GO enrichment of DEG sets for mixture treatments. Table shows all GO terms with significant enrichment (adjusted p-value < 0.05) across specific or non-specific DEG sets. Color indicates –log_10_(p-value)

**Table S6** Comparison of GO enrichment within kmeans clusters. Table shows all GO terms with significant enrichment (adjusted p-value < 0.05) across clusters. Color indicates –log_10_(p-value)

**Table S7** Results of differential gene expression for *Abbc3*, *Aldh1a1*, *Cyp3a23/3a1*, and *Mme* measured by q-PCR analysis

| Treatment | Dose | *Abcc3* | | *Aldh1a1* | | *Cyp3a23/3a1* | | *Mme* | |
| --- | --- | --- | --- | --- | --- | --- | --- | --- | --- |
|  |  | Mean | SD | Mean | SD | Mean | SD | Mean | SD |
| Control | - | 1.04 | 0.32 | 1.05 | 0.35 | 1.03 | 0.26 | 1.03 | 0.25 |
| Thiacloprid [mg/kg BW/day] | 10 | 2.60 | 0.23 | 1.43 | 0.29 | 2.45 | 0.60 | 2.48 | 0.69 |
|  | 43 | 3.21 | 1.51 | 1.54 | 0.86 | 2.49 | 1.09 | 4.46 | 2.08 |
|  | 75 | 7.95 | 1.40 | 3.16 | 0.77 | 10.03 | 4.13 | 16.58 | 9.63 |
|  | 108 | 7.95 | 1.67 | 3.16 | 1.16 | 10.03 | 4.14 | 16.58 | 7.86 |
|  | 140 | 25.52 | 7.12 | 8.47 | 1.80 | 65.40 | 13.68 | 50.30 | 14.11 |
| Imazalil [mg/kg BW/day] | 10 | 1.63 | 0.24 | 0.74 | 0.44 | 1.47 | 0.34 | 1.37 | 0.15 |
|  | 38 | 6.40 | 2.83 | 3.23 | 2.62 | 13.01 | 9.30 | 4.97 | 4.88 |
|  | 65 | 7.71 | 1.74 | 3.50 | 1.92 | 20.22 | 10.94 | 11.63 | 5.36 |
|  | 93 | 11.63 | 4.95 | 2.80 | 0.13 | 27.27 | 7.08 | 10.72 | 6.13 |
|  | 120 | 10.54 | 2.15 | 4.21 | 0.78 | 31.85 | 6.43 | 20.52 | 7.64 |
| Clothianidin [mg/kg BW/day] | 100 | 4.57 | 2.09 | 4.60 | 2.34 | 5.91 | 2.48 | 2.92 | 0.88 |
|  | 163 | 5.82 | 2.50 | 9.83 | 7.67 | 10.21 | 5.93 | 11.17 | 4.71 |
|  | 225 | 6.67 | 4.71 | 16.51 | 10.80 | 13.50 | 7.02 | 14.32 | 8.06 |
|  | 288 | 8.16 | 5.66 | 11.42 | 11.68 | 7.00 | 1.28 | 6.64 | 1.92 |
|  | 350 | 11.51 | 10.24 | 11.95 | 14.09 | outlier | - | 6.65 | 1.61 |
| Thiacloprid + Imazalil [mg/kg BW/day] | 5 + 4 | 2.44 | 1.27 | 5.26 | 3.23 | 4.44 | 2.43 | 2.24 | 0.63 |
|  | 21 + 18 | 2.47 | 1.92 | 4.47 | 3.12 | 4.02 | 1.85 | 0.59 | 0.60 |
|  | 38 + 32 | 3.80 | 1.16 | 2.20 | 0.26 | 8.67 | 2.78 | 6.28 | 3.23 |
|  | 54 + 46 | 6.80 | 4.26 | 6.26 | 2.49 | 18.22 | 11.89 | 8.41 | 5.08 |
|  | 70 + 60 | 8.34 | 1.05 | 9.55 | 4.49 | 42.18 | 24.72 | 13.85 | 5.83 |
| Thiacloprid + Clothianidin [mg/kg BW/day] | 5 + 13 | 0.81 | 0.07 | 0.65 | 0.01 | 1.35 | 0.32 | 0.83 | 0.12 |
|  | 21 + 53 | 2.73 | 1.56 | 2.55 | 1.47 | 3.68 | 1.39 | 29.86 | 31.11 |
|  | 38 + 94 | 8.99 | 0.64 | 11.88 | 6.93 | 19.88 | 5.77 | 17.22 | 4.33 |
|  | 54 + 134 | 8.01 | 6.72 | 10.93 | 6.94 | 26.87 | 16.92 | 34.45 | 22.55 |
|  | 70 + 175 | 8.48 | 2.52 | 8.30 | 2.23 | 33.37 | 6.63 | 20.86 | 9.67 |
| Imazalil + Clothianidin [mg/kg BW/day] | 5 + 15 | 1.12 | 0.52 | 1.43 | 1.23 | 2.06 | 1.51 | 0.85 | 0.56 |
|  | 19 + 55 | 3.41 | 3.59 | 1.04 | 0.71 | 4.71 | 2.19 | 1.56 | 1.36 |
|  | 33 + 95 | 4.61 | 3.06 | 4.88 | 4.12 | 11.23 | 8.45 | 5.75 | 4.01 |
|  | 46 + 135 | 5.06 | 2.09 | 3.38 | 1.89 | 17.93 | 10.91 | 21.18 | 21.43 |
|  | 60 + 175 | 6.63 | 1.84 | 4.68 | 1.51 | 25.29 | 16.48 | 14.39 | 8.08 |


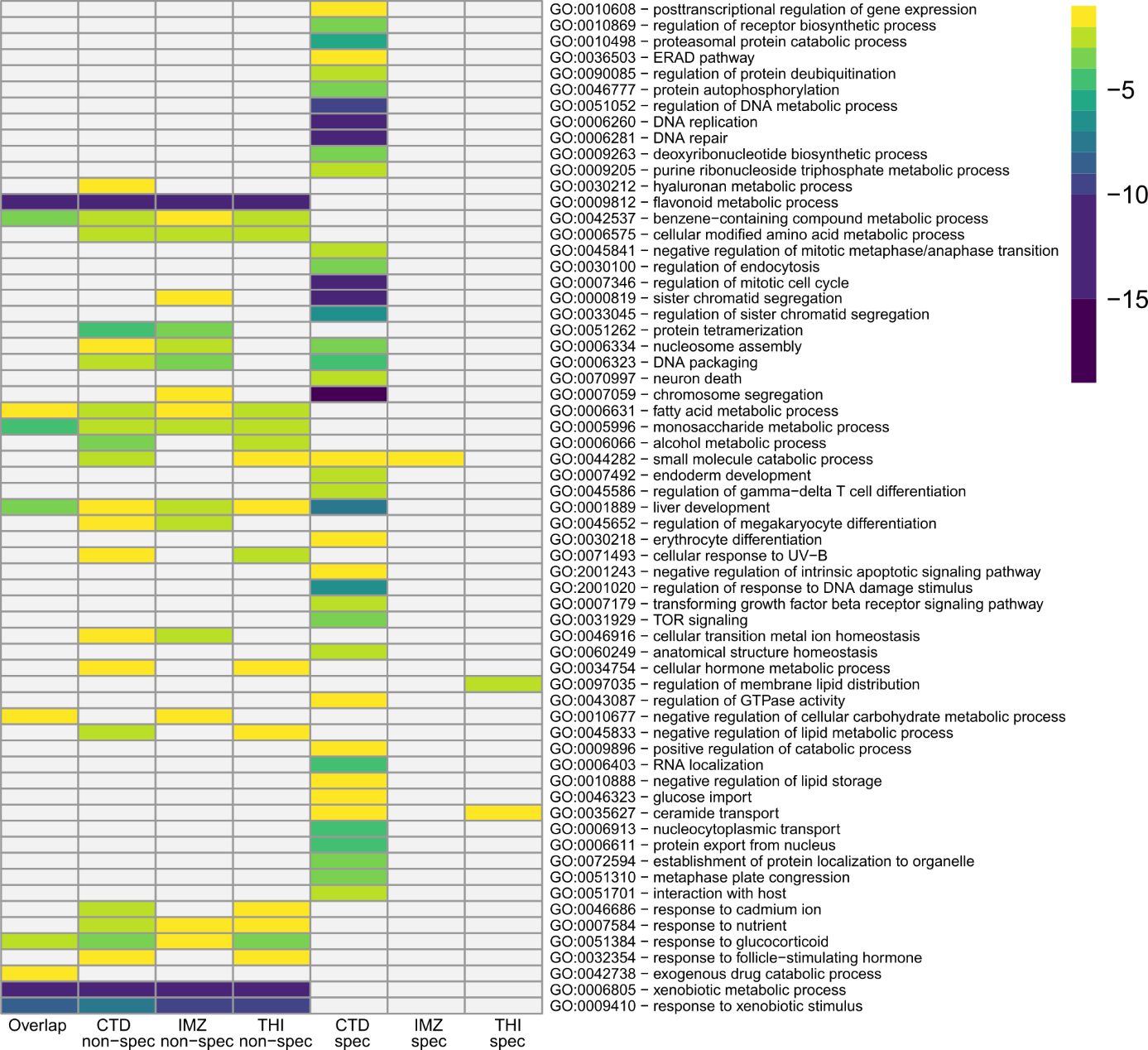


**Fig. S1** Comparison of GO enrichment of DEG sets for single compound treatments. Heatmap shows selected GO terms with significant enrichment (adjusted p-value < 0.05) across specific or non-specific DEG sets. Color indicates –log_10_(p-value)


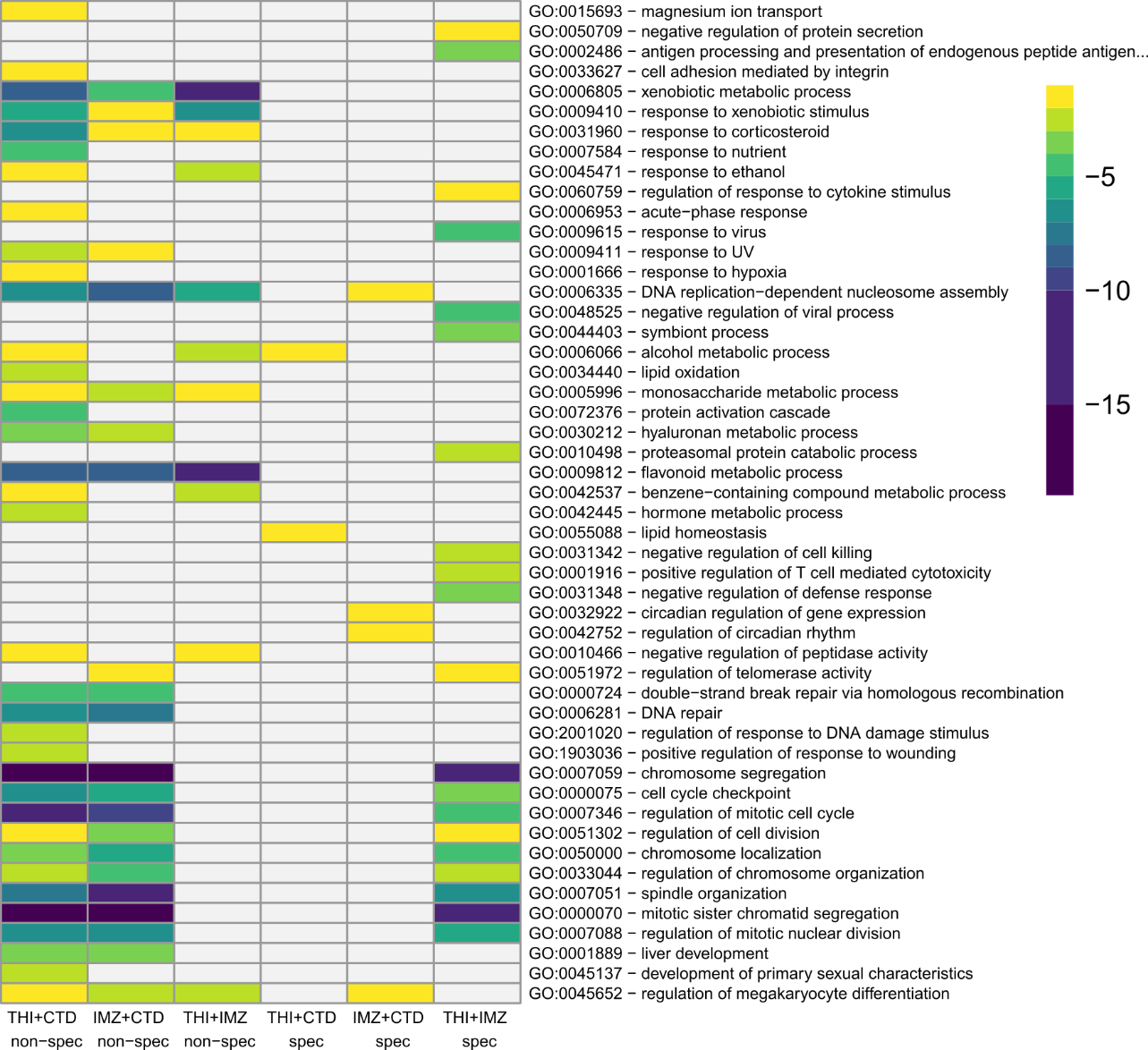


**Fig. S2** Comparison of GO enrichment of DEG sets for mixture treatments. Heatmap shows selected GO terms with significant enrichment (adjusted p-value < 0.05) across specific or non-specific DEG sets. Color indicates –log_10_(p-value)


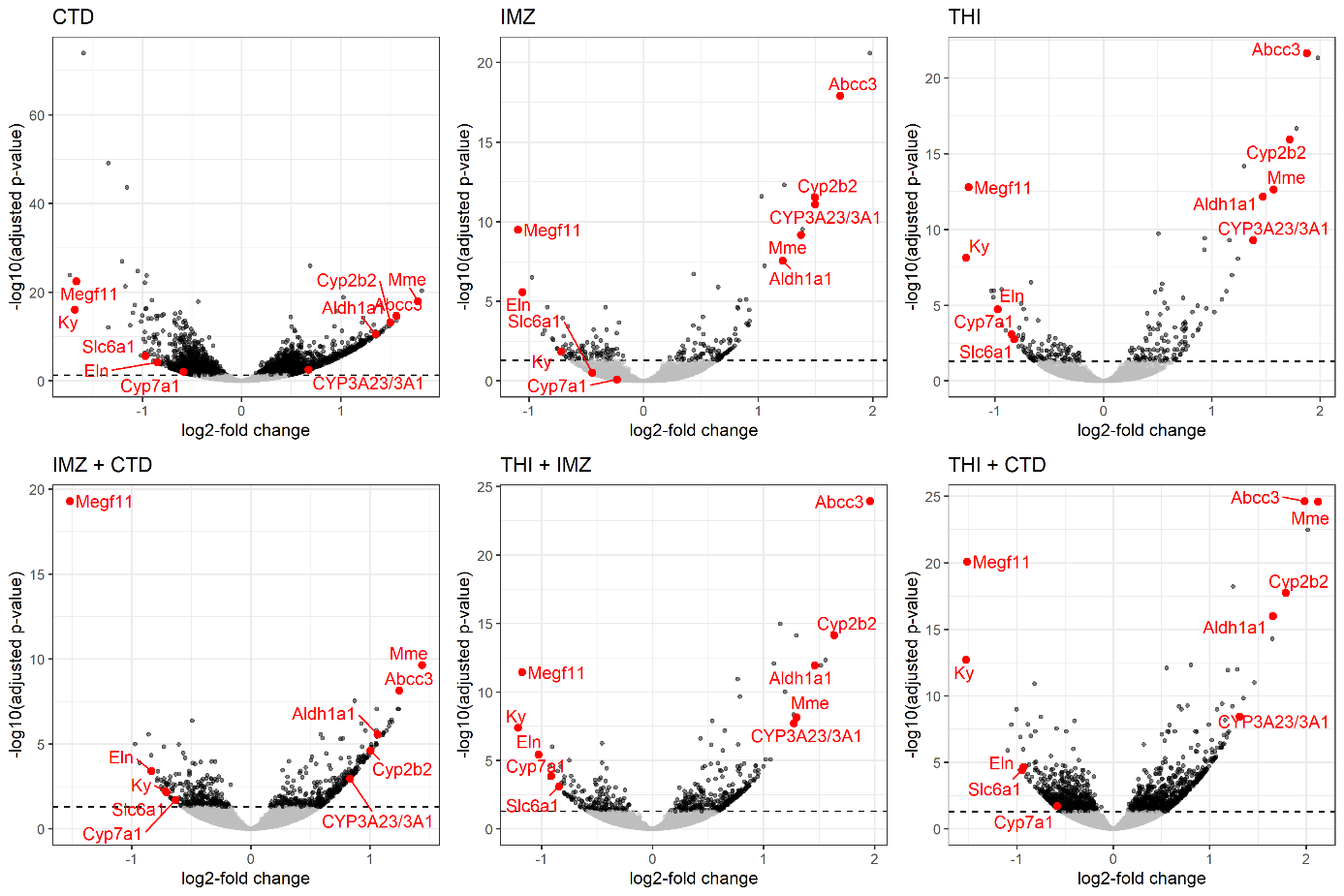


**Fig. S3** Volcano plots of transcriptional changes for each comparison. Black circles indicate significantly changed genes (adjusted p-value < 0.05, dashed line) and grey circles show non significantly changed genes. Red circles and text highlight the selected top ten DGE

**Fig. S4** Validation of top most regulated genes in rat hepatocytes by qRT-PCR. mRNA was transcribed into cDNA and qRT-PCR was performed. Data are presented as means of rat biological samples. Fold change value >1 represents an upregulation while fold change value <0 represents a downregulation. Fold change value >40 is indicated by “a” while fold change value < -15 is indicated by “b”. Differences between means were determined by the nonparametric Kruskal-Wallis test followed by Dunn's test (*p < 0.05; **p < 0.01; ***p < 0.001). When Dunn's test was not applicable, Dunnett's test was run instead


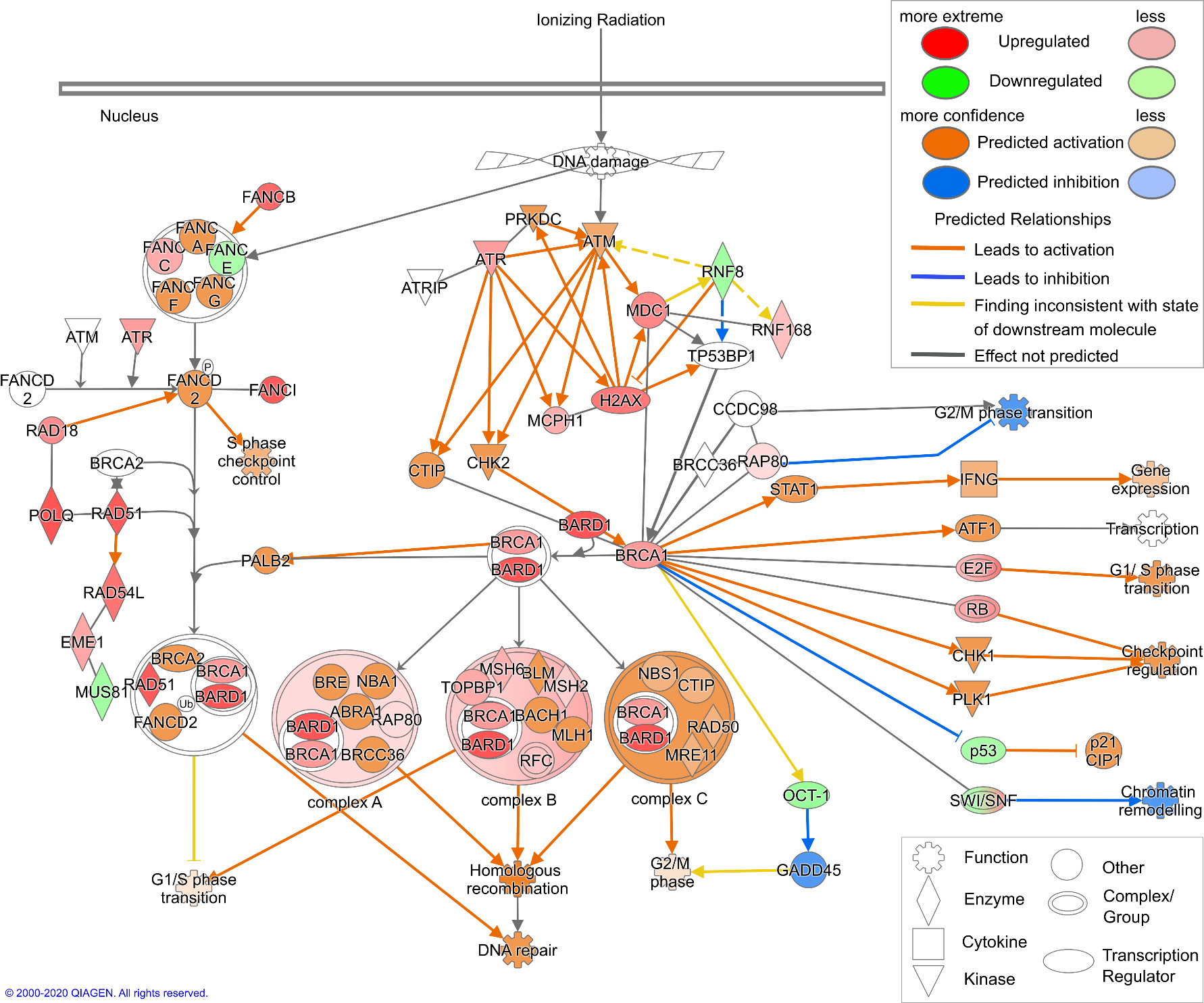


**Fig. S5** Network of DNA repair response elicited specifically by CTD in rat liver, as computed with Ingenuity Pathway Analysis software. Continuous lines indicate direct functional relationships, dashed lines indicate indirect connections. Network nodes are labeled with the gene name and the functional classification of genes is denoted by their different shapes. Additional nodes were added to the network manually based on literature research (Bonner et al. 2008; Hustedt and Durocher 2016)

**Fig. S6** Representative dose-response modeling of gene expression. The curves represent the four-parameter exponential model. For testing of mixture effects, the dose-response data for the single compounds and mixtures were compared using the benchmark dose modeling software PROAST. Data are shown as means and SD. The concentration-response of the mixture (green diamonds) shows no derivation from the overall concentration-response fit, indicating that dose addition can be assumed

*****

******

*******

******

******

*******

******

******

*******

*****

******

******

*****

******

*****

*****

*****

*****

******

*****

a

a

*****

******

*******

*****

******

******

******

******

******

*****

******

*****

******

******

******

*****

*****

a

*****

******

******

*****

*****

*****

*******

*******

*****

*****

******

*******

*****

******

******

*****

*****

*****

*****

*****

*****

*****

*****

*****

*****

*****

******

*****

******

*****

******

*****

*****

*****

*****

*****

b

*****

******

*****

******

*******

*****

*****

******

*****

*****

******

******

*****

*****

******

*****

*****

*****

******

*******

*****

*****

*****

*****

*****

*****

******

*****


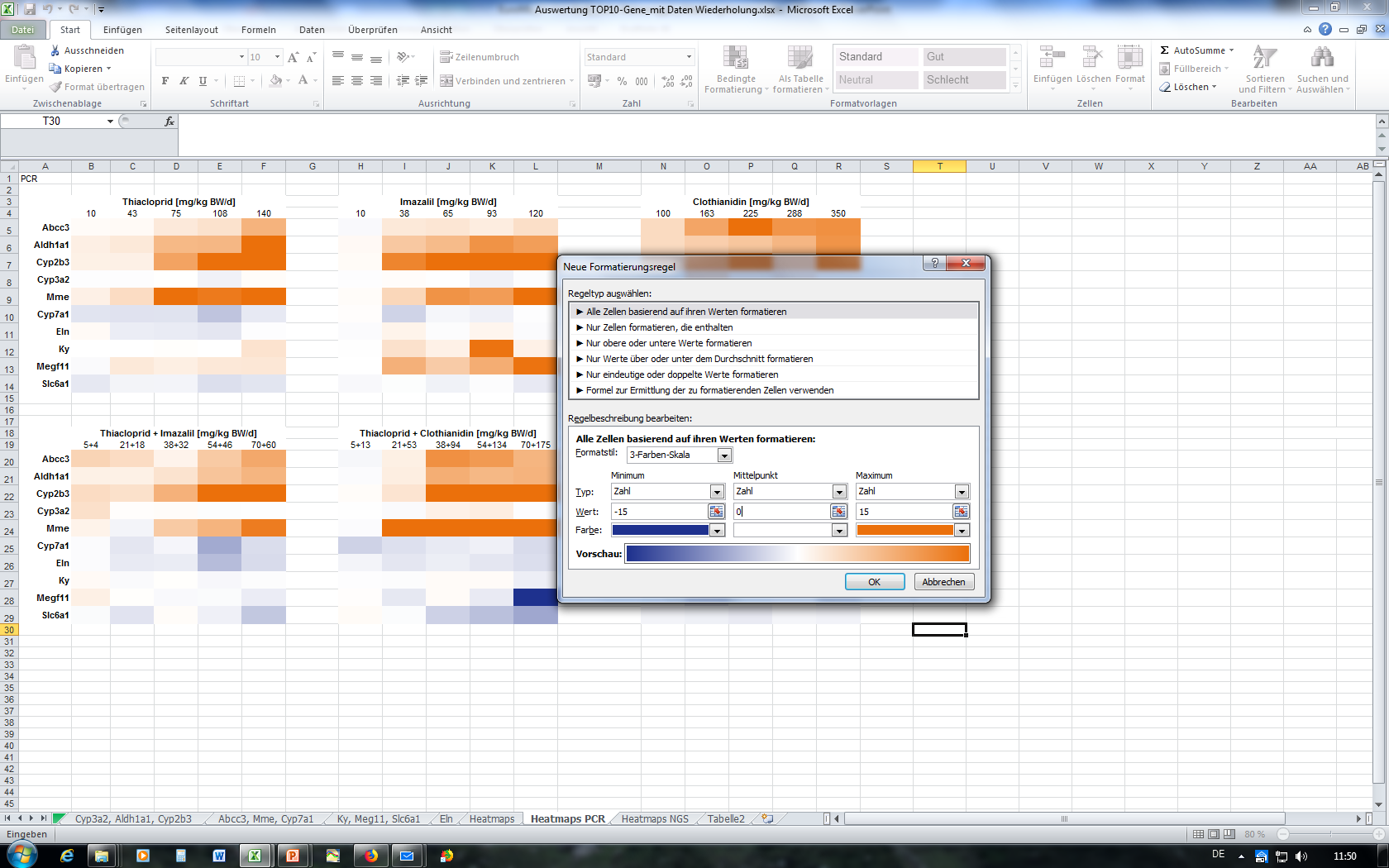


**-15**

**40**

**Fold change**

IMZ

THI

Mix

Log dose equivalents [mg/kg BW]


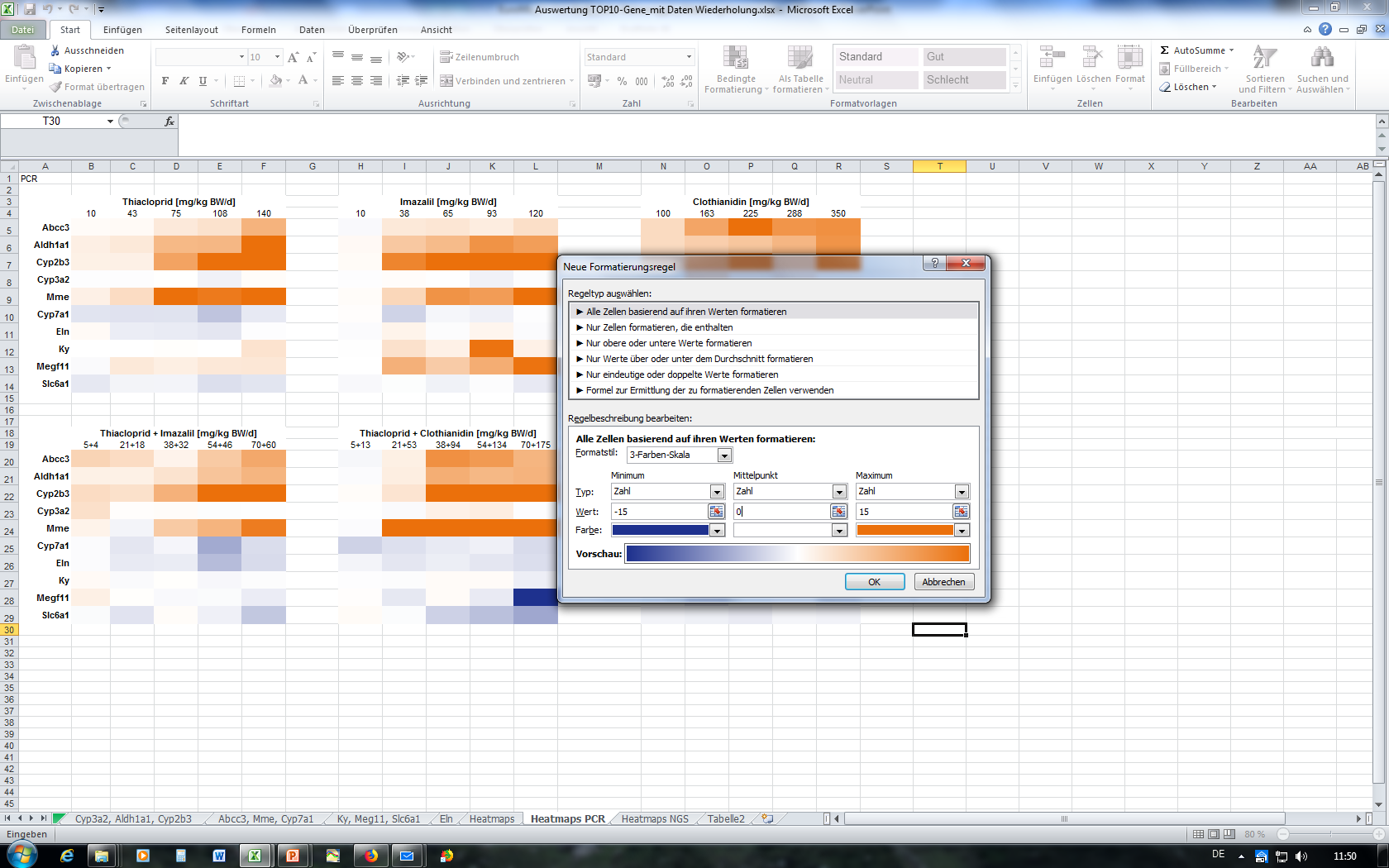


CTD

IMZ

Mix

Log dose equivalents [mg/kg BW]

CTD

THI

Mix

Log dose equivalents [mg/kg BW]

Abcc3 relative expression


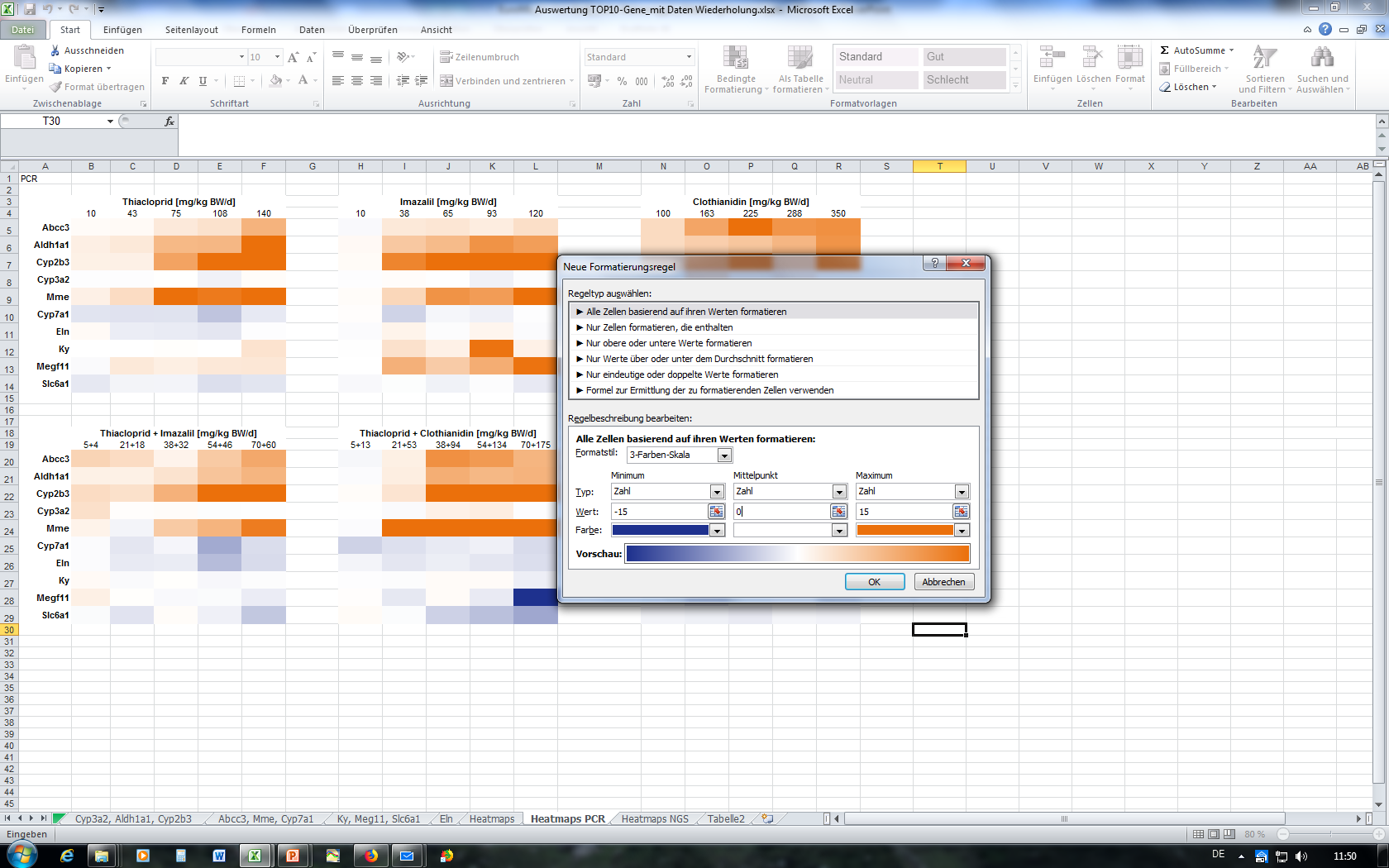


IMZ

THI

Mix

Log dose equivalents [mg/kg BW]

CTD

IMZ

Mix

Log dose equivalents [mg/kg BW]


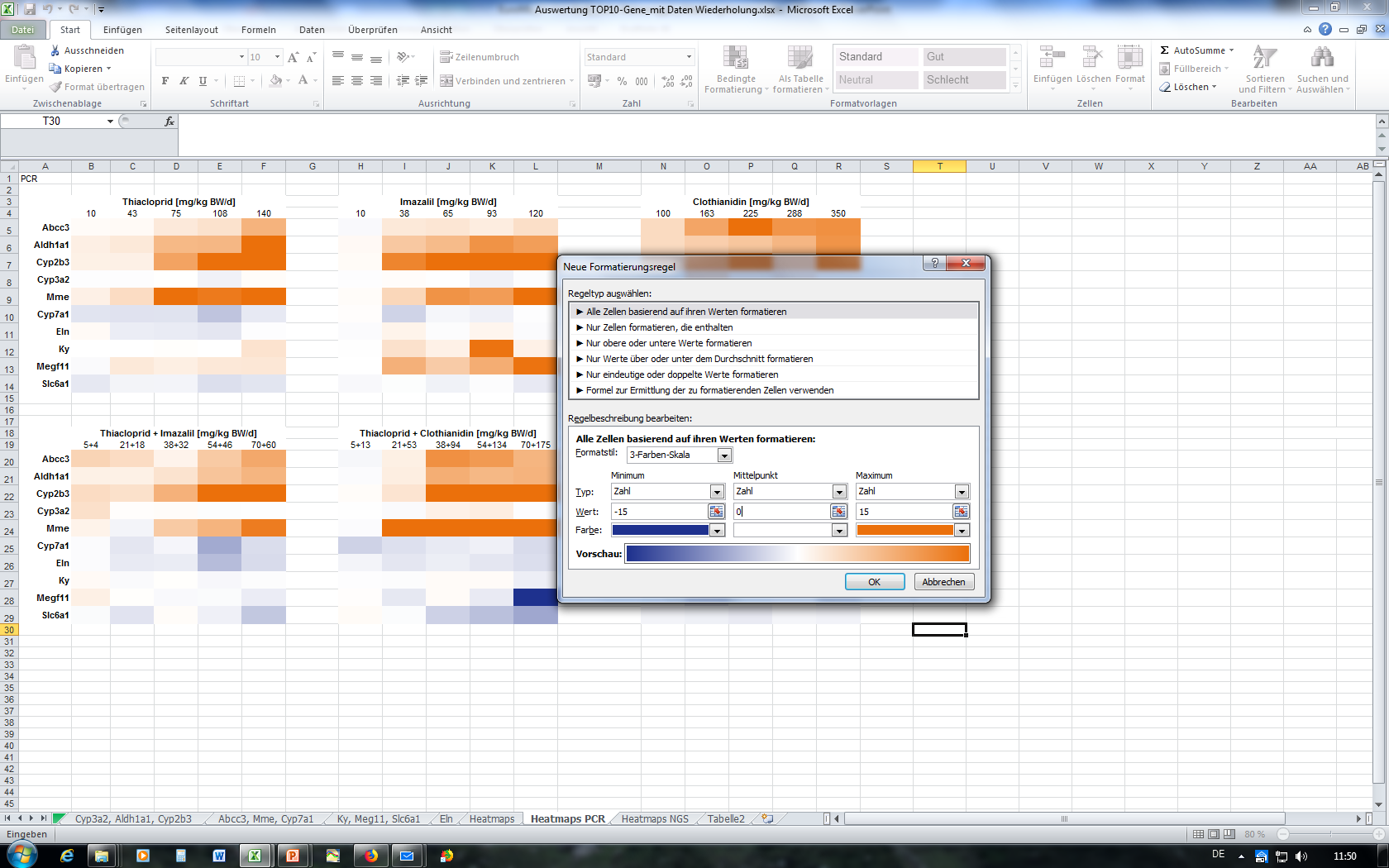


CTD

THI

Mix

Log dose equivalents [mg/kg BW]

Aldh1a1 relative expression

CTD

THI

Mix

Log dose equivalents [mg/kg BW]

Abcc3 relative expression


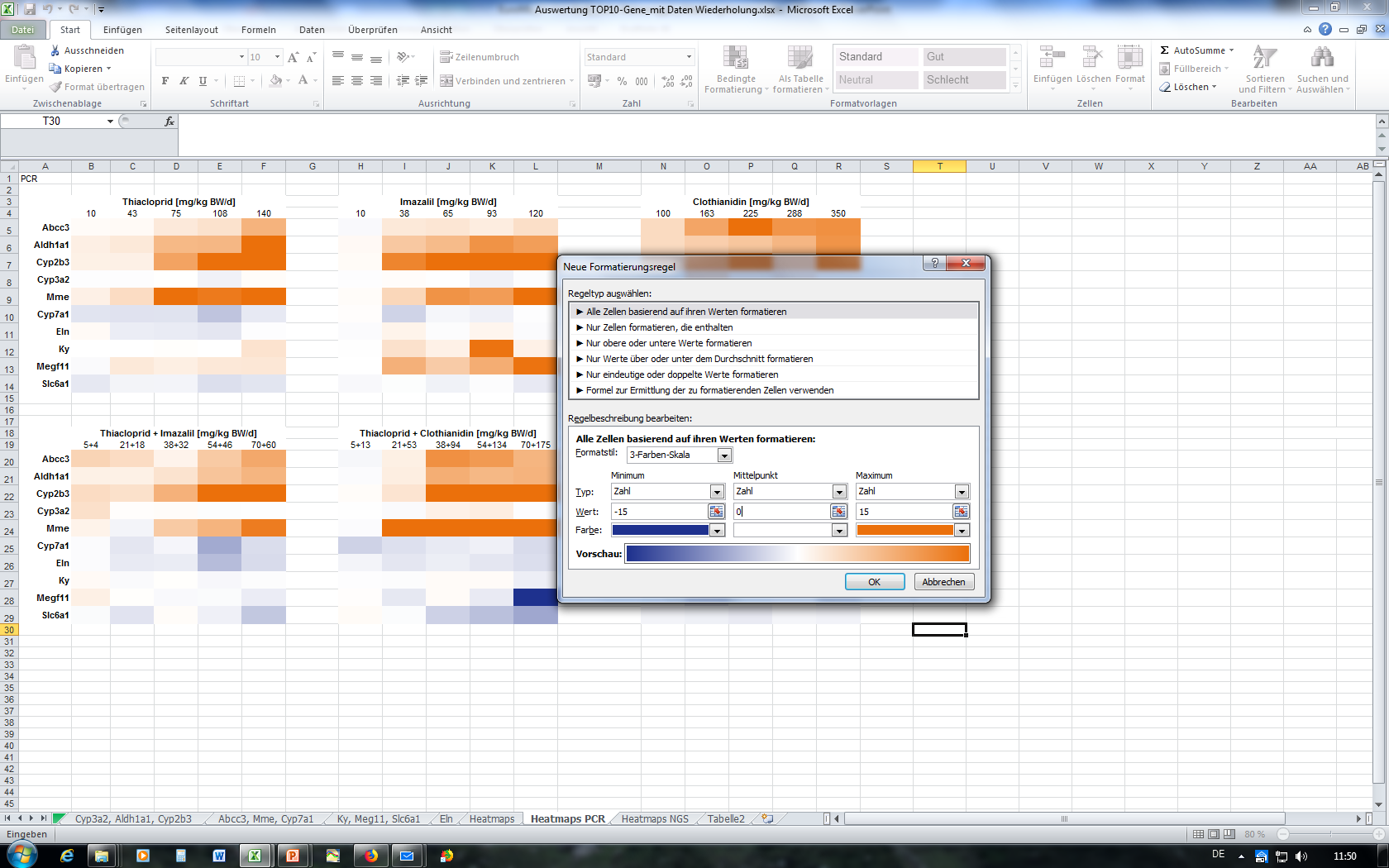


CTD

IMZ

Mix

Log dose equivalents [mg/kg BW]

IMZ

THI

Mix

Log dose equivalents [mg/kg BW]


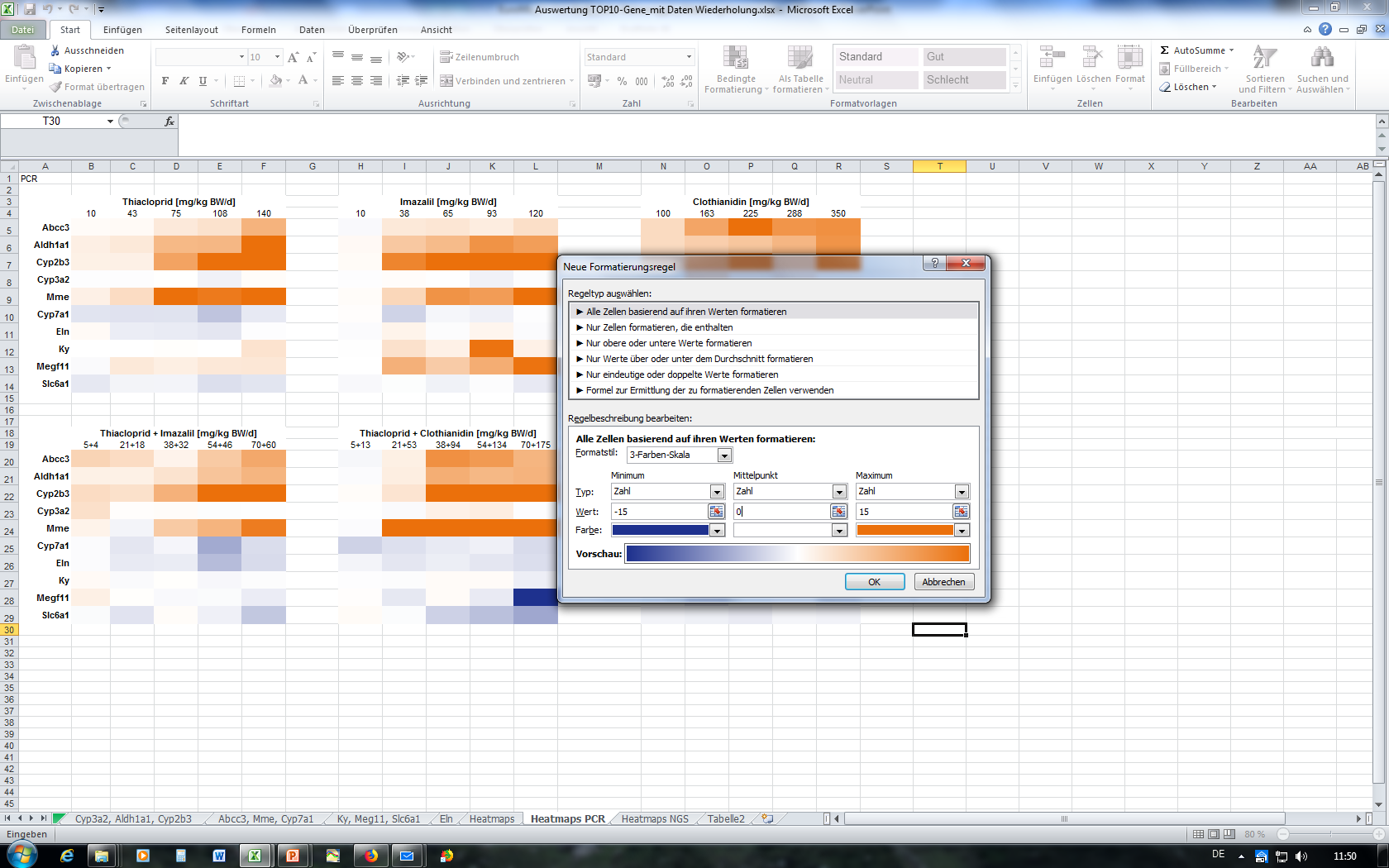


CTD

THI

Mix

Log dose equivalents [mg/kg BW]

Mme relative expression

IMZ

THI

Mix

Log dose equivalents [mg/kg BW]

CTD

IMZ

Mix

Log dose equivalents [mg/kg BW]


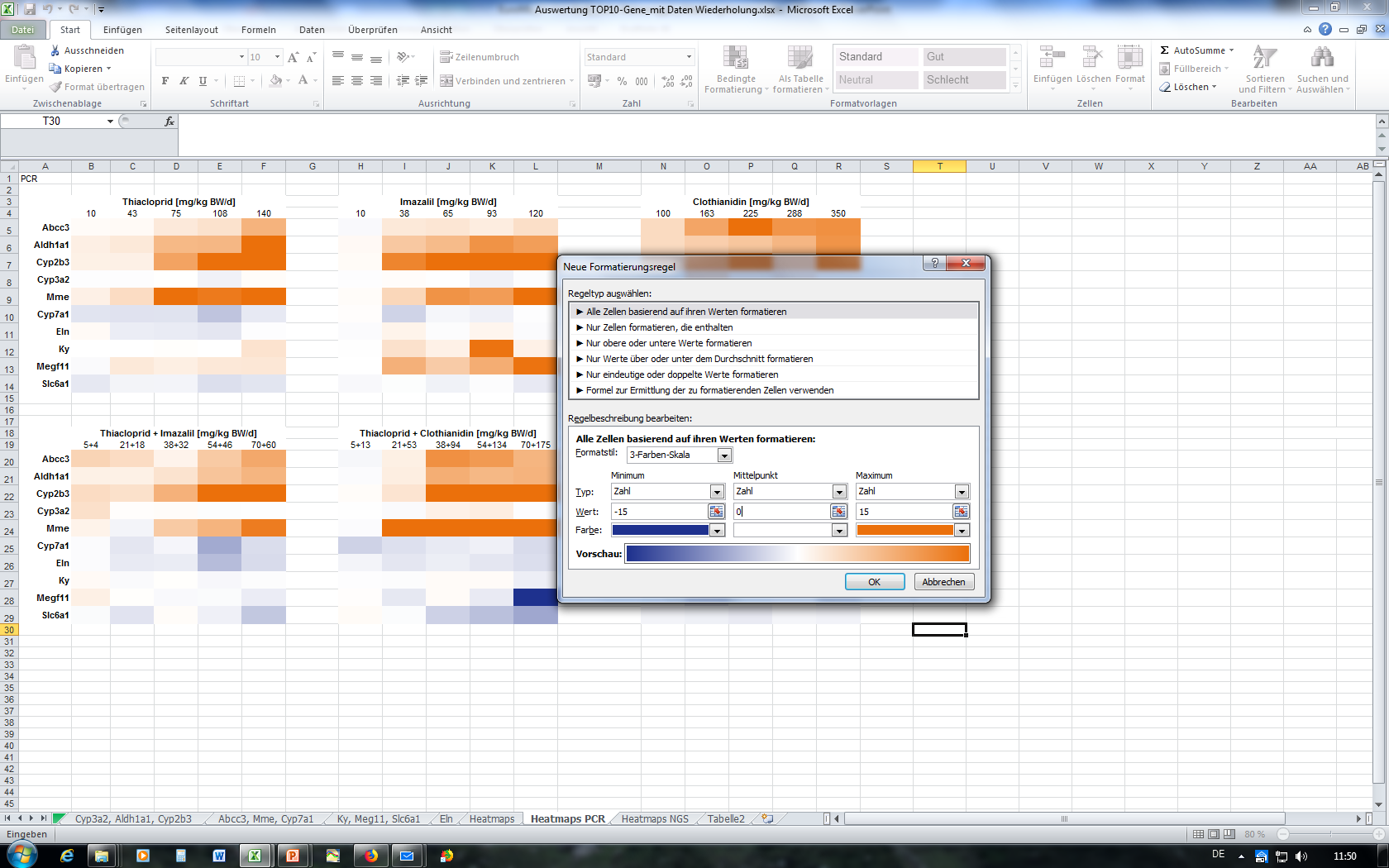


**Fig. S6 (continued)**


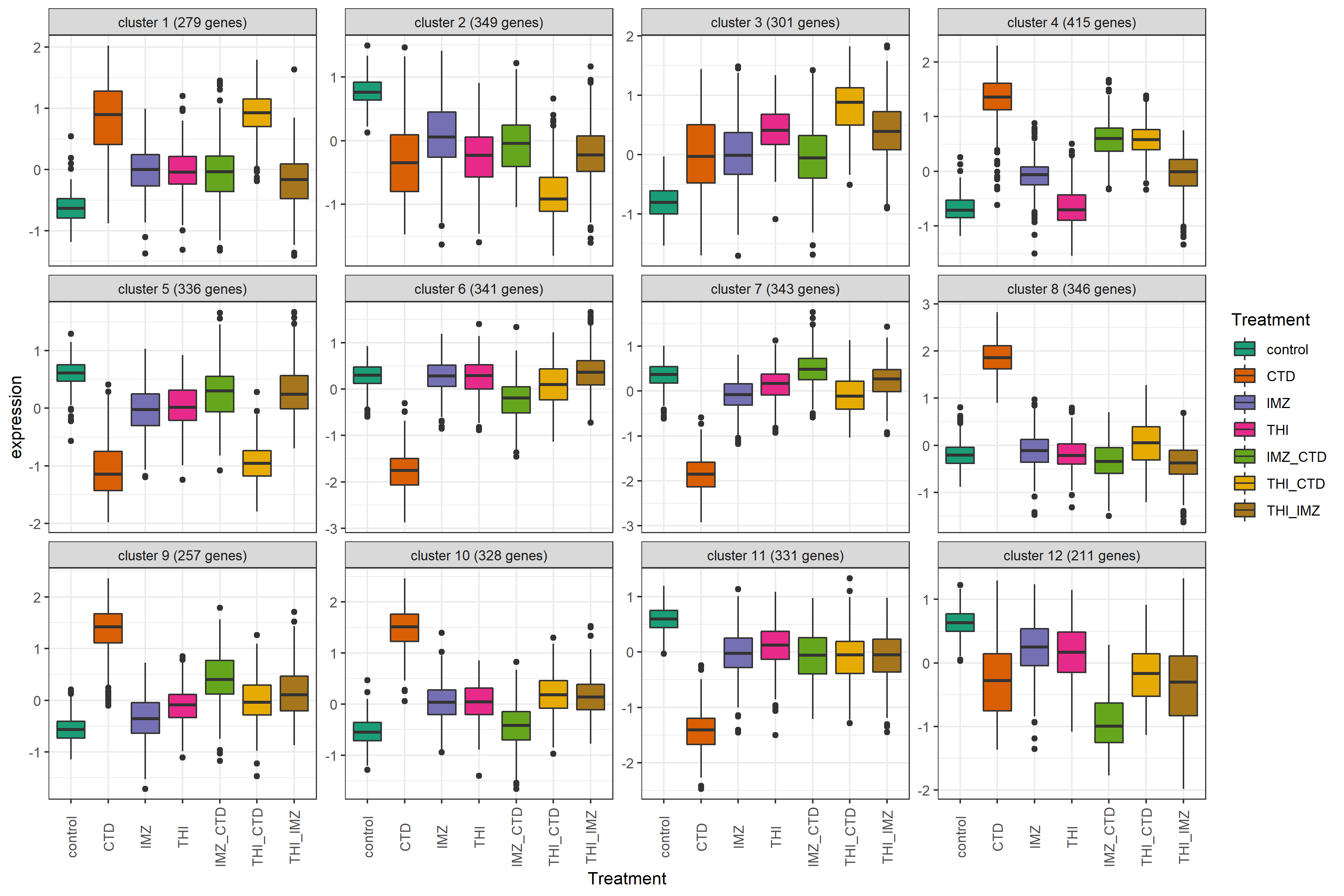


**Fig. S7** Boxplot of scaled gene expression per treatment per cluster. All 3837 DEGs were grouped by kmeans clustering into twelve clusters


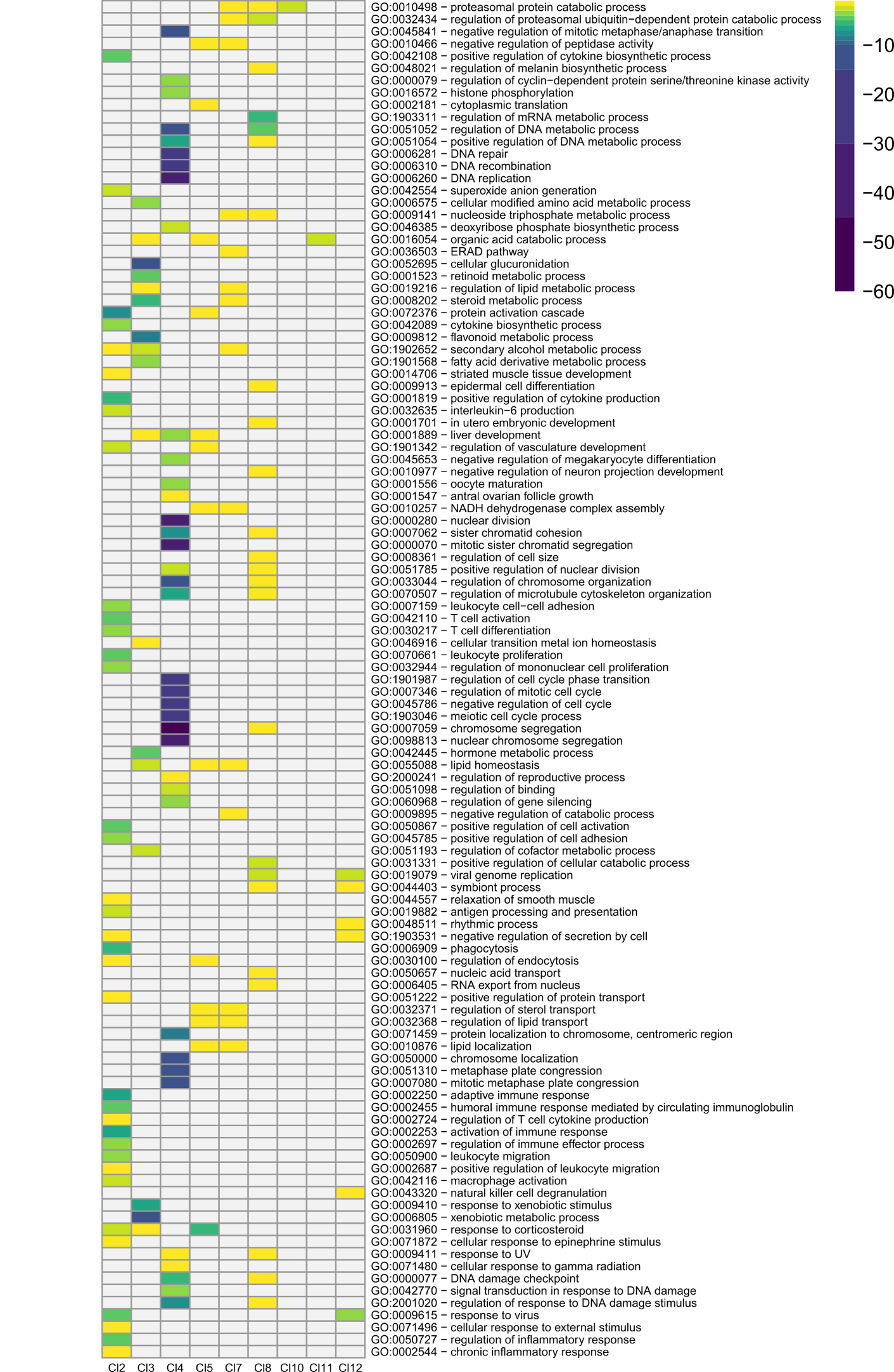


**Fig. S8** Comparison of GO enrichment within kmeans clusters. Heatmap shows selected GO terms with significant enrichment (adjusted p-value < 0.05) across clusters. Color indicates –log_10_(p-value). Cluster 1, 6 and 9 are not shown


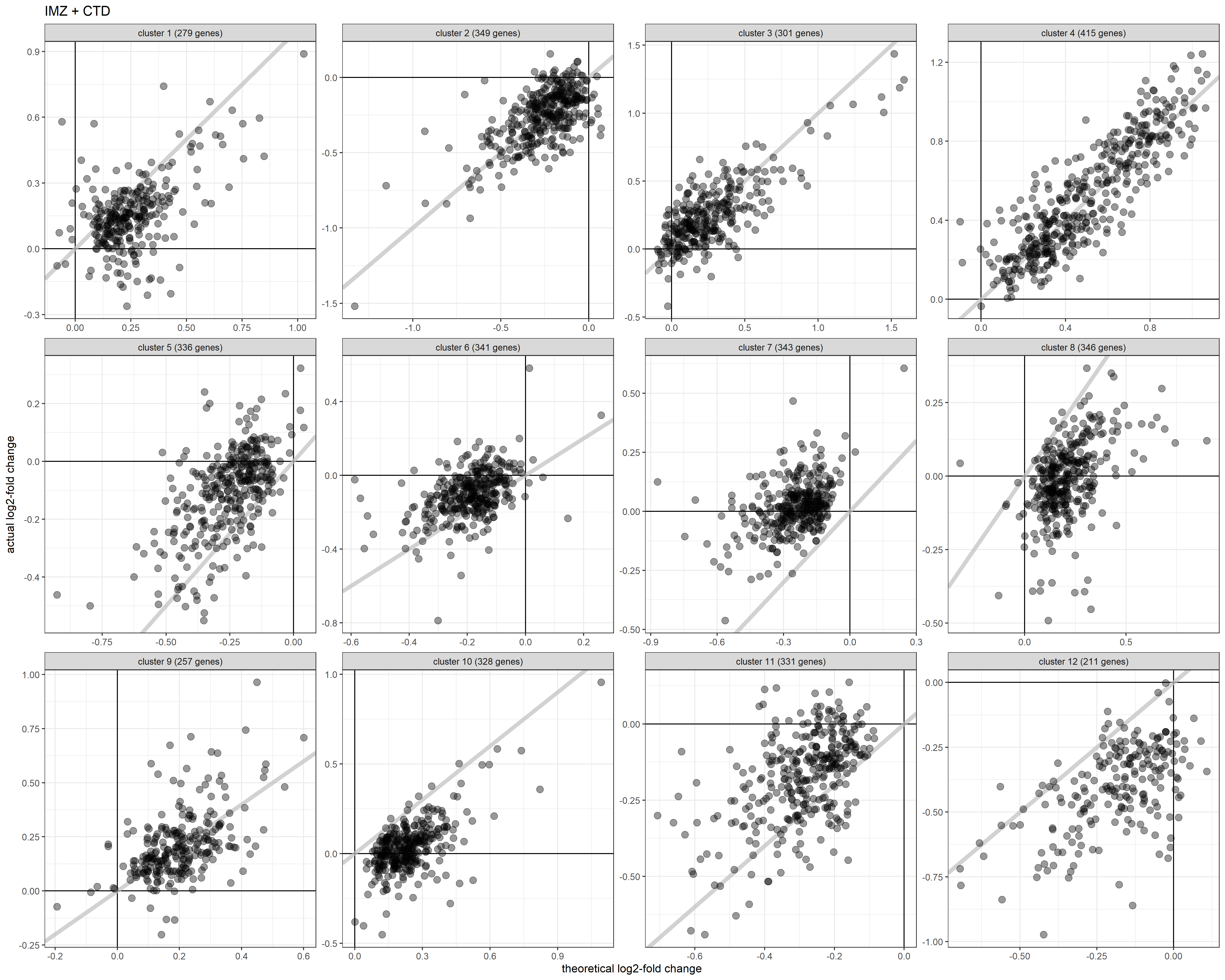


**Fig. S9** Scatterplot of actual versus theoretical log_2_-fold change for genes grouped by kmeans clustering for IMZ+CTD. Grey line indicates the theoretical additive response of mixture


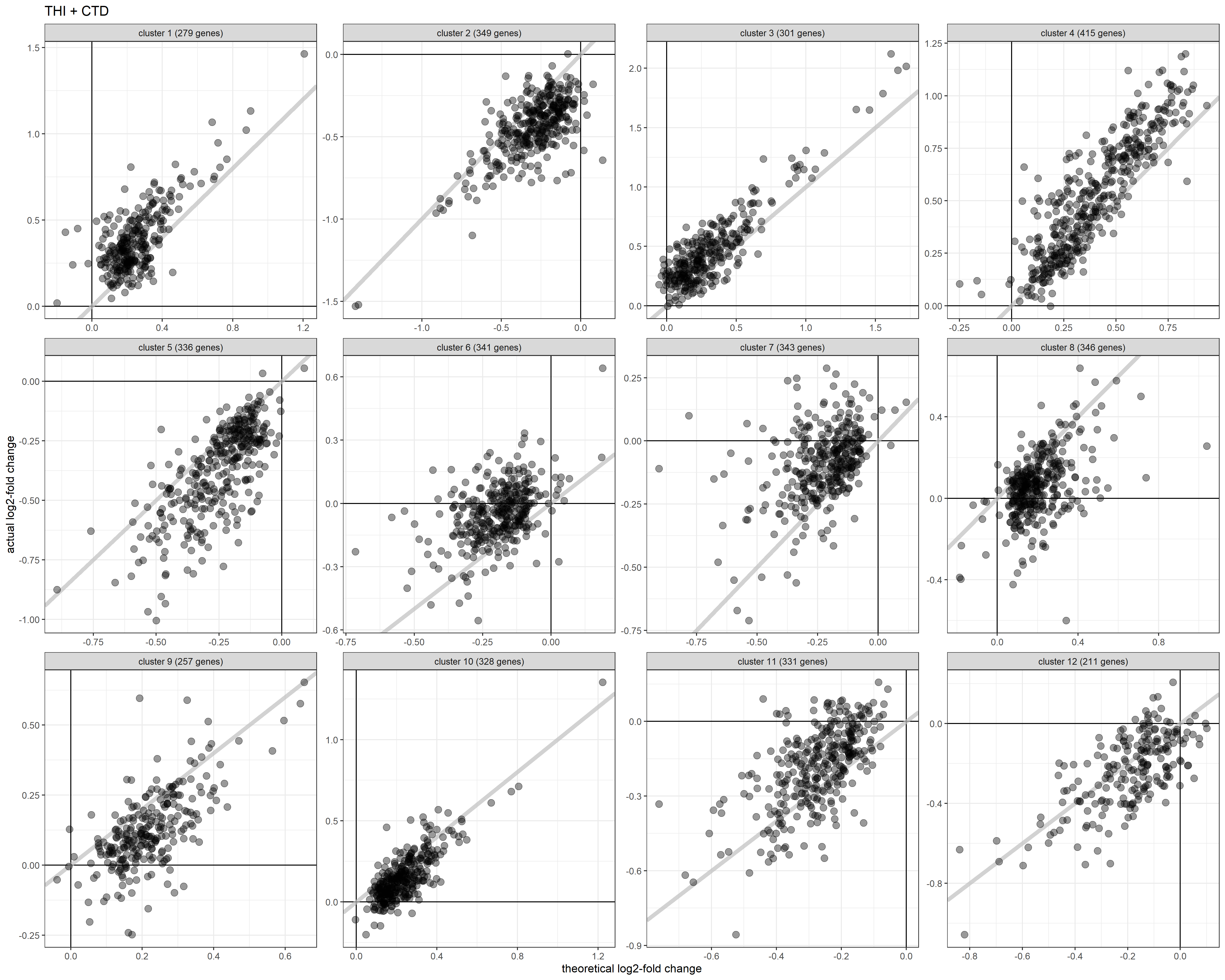


**Fig. S10** Scatterplot of actual versus theoretical log_2_-fold change for genes grouped by kmeans clustering for THI+CTD. Grey line indicates the theoretical additive response of mixture


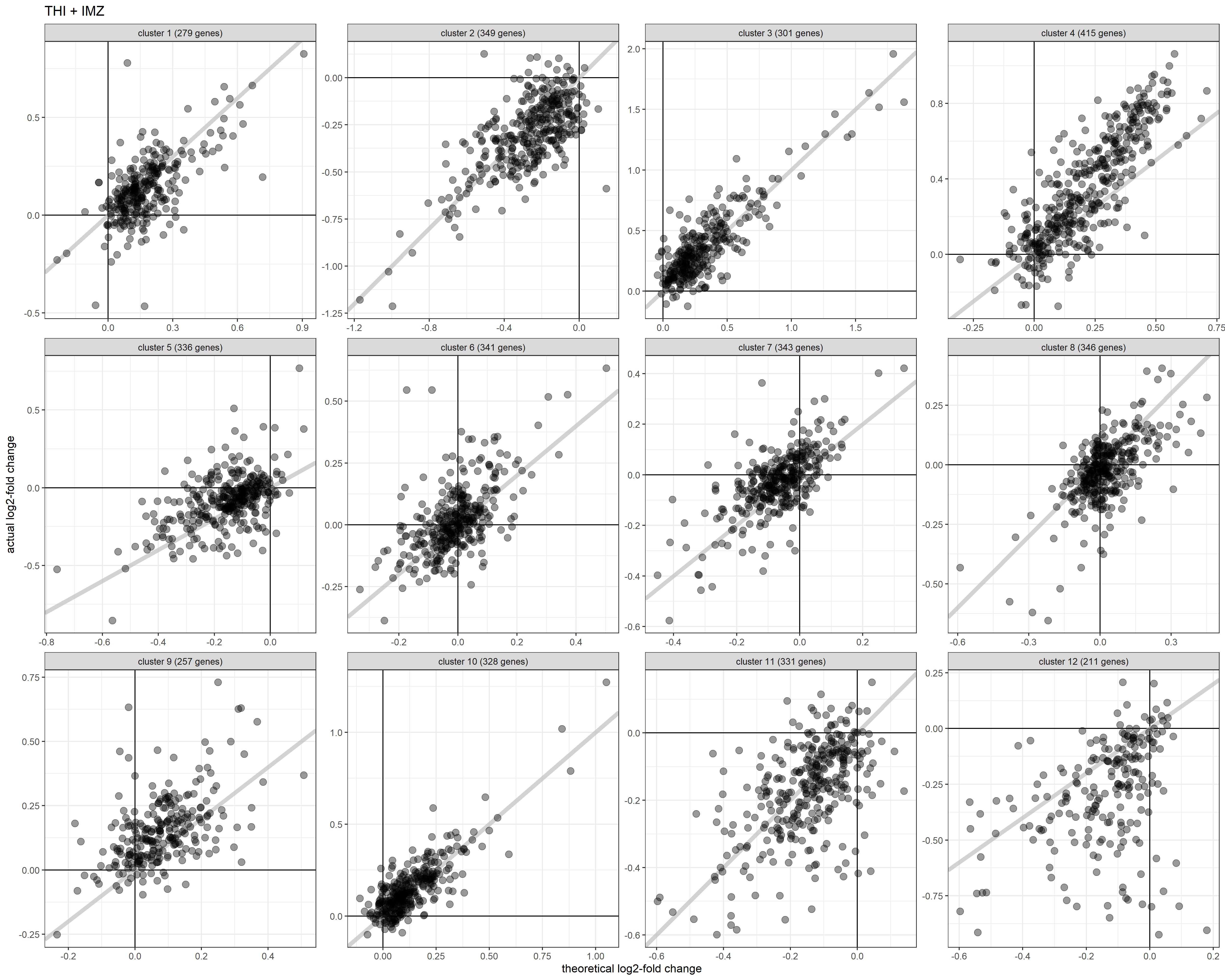


**Fig. S11** Scatterplot of actual versus theoretical log_2_-fold change for genes grouped by kmeans clustering for THI+IMZ. Grey line indicates the theoretical additive response of mixture
